# The effect of a high-polyphenol Mediterranean diet (Green-MED) combined with physical activity on age-related brain atrophy: the Dietary Intervention Randomized Controlled Trial Polyphenols Unprocessed Study (DIRECT PLUS)

**DOI:** 10.1093/ajcn/nqac001

**Published:** 2022-01-11

**Authors:** Alon Kaplan, Hila Zelicha, Anat Yaskolka Meir, Ehud Rinott, Gal Tsaban, Gidon Levakov, Ofer Prager, Moti Salti, Yoram Yovell, Jonathan Ofer, Sebastian Huhn, Frauke Beyer, Veronica Witte, Arno Villringer, Nachshon Meiran, Tamar B Emesh, Peter Kovacs, Martin von Bergen, Uta Ceglarek, Matthias Blüher, Michael Stumvoll, Frank B Hu, Meir J Stampfer, Alon Friedman, Ilan Shelef, Galia Avidan, Iris Shai

**Affiliations:** The Health & Nutrition Innovative International Research Center, Faculty of Health Sciences, Ben-Gurion University of the Negev, Beer-Sheva, Israel; The Health & Nutrition Innovative International Research Center, Faculty of Health Sciences, Ben-Gurion University of the Negev, Beer-Sheva, Israel; The Health & Nutrition Innovative International Research Center, Faculty of Health Sciences, Ben-Gurion University of the Negev, Beer-Sheva, Israel; The Health & Nutrition Innovative International Research Center, Faculty of Health Sciences, Ben-Gurion University of the Negev, Beer-Sheva, Israel; The Health & Nutrition Innovative International Research Center, Faculty of Health Sciences, Ben-Gurion University of the Negev, Beer-Sheva, Israel; Soroka University Medical Center, Beer-Sheva, Israel; Department of Cognitive and Brain Sciences, Ben-Gurion University of the Negev, Beer-Sheva, Israel; Zlotowski Center for Neuroscience, Ben-Gurion University of the Negev, Beer-Sheva, Israel; Zlotowski Center for Neuroscience, Ben-Gurion University of the Negev, Beer-Sheva, Israel; Zlotowski Center for Neuroscience, Ben-Gurion University of the Negev, Beer-Sheva, Israel; Department of Medical Neurobiology, The Hebrew University-Hadassah School of Medicine, Jerusalem, Israel; Zlotowski Center for Neuroscience, Ben-Gurion University of the Negev, Beer-Sheva, Israel; Helmholtz Centre for Environmental Research (UFZ), Leipzig, Germany; Department of Neurology, Max-Planck-Institute for Human Cognitive and Brain Sciences, and Cognitive Neurology, University of Leipzig Medical Center, Leipzig, Germany; Department of Neurology, Max-Planck-Institute for Human Cognitive and Brain Sciences, and Cognitive Neurology, University of Leipzig Medical Center, Leipzig, Germany; Department of Neurology, Max-Planck-Institute for Human Cognitive and Brain Sciences, and Cognitive Neurology, University of Leipzig Medical Center, Leipzig, Germany; Department of Cognitive and Brain Sciences, Ben-Gurion University of the Negev, Beer-Sheva, Israel; Department of Cognitive and Brain Sciences, Ben-Gurion University of the Negev, Beer-Sheva, Israel; Integrated Research and Treatment Center (IFB) Adiposity Diseases, Leipzig University Medical Center, Leipzig, Germany; Department of Molecular Systems Biology, Helmholtz Centre for Environmental Research (UFZ), Leipzig, Germany; Institute of Laboratory Medicine, Clinical Chemistry and Molecular Diagnostics, University Hospital Leipzig, Leipzig, Germany; Department of Medicine, Leipzig University, Leipzig, Germany; Department of Medicine, Leipzig University, Leipzig, Germany; Department of Nutrition, Harvard TH Chan School of Public Health, Boston, MA, USA; Department of Medicine, Harvard Channing Division of Network Medicine, Brigham and Women's Hospital, Boston, MA, USA; Department of Nutrition, Harvard TH Chan School of Public Health, Boston, MA, USA; Department of Medicine, Harvard Channing Division of Network Medicine, Brigham and Women's Hospital, Boston, MA, USA; Zlotowski Center for Neuroscience, Ben-Gurion University of the Negev, Beer-Sheva, Israel; Department of Medical Neuroscience, Faculty of Medicine, Dalhousie University, Halifax, Nova Scotia, Canada; The Health & Nutrition Innovative International Research Center, Faculty of Health Sciences, Ben-Gurion University of the Negev, Beer-Sheva, Israel; Soroka University Medical Center, Beer-Sheva, Israel; Department of Cognitive and Brain Sciences, Ben-Gurion University of the Negev, Beer-Sheva, Israel; Zlotowski Center for Neuroscience, Ben-Gurion University of the Negev, Beer-Sheva, Israel; The Health & Nutrition Innovative International Research Center, Faculty of Health Sciences, Ben-Gurion University of the Negev, Beer-Sheva, Israel; Department of Nutrition, Harvard TH Chan School of Public Health, Boston, MA, USA

**Keywords:** polyphenols, dietary intervention, Green Mediterranean diet, aging, age-related atrophy, neurodegeneration, hippocampal occupancy score

## Abstract

**Background:**

The effect of diet on age-related brain atrophy is largely unproven.

**Objectives:**

We aimed to explore the effect of a Mediterranean diet (MED) higher in polyphenols and lower in red/processed meat (Green-MED diet) on age-related brain atrophy.

**Methods:**

This 18-mo clinical trial longitudinally measured brain structure volumes by MRI using hippocampal occupancy score (HOC) and lateral ventricle volume (LVV) expansion score as neurodegeneration markers. Abdominally obese/dyslipidemic participants were randomly assigned to follow 1) healthy dietary guidelines (HDG), 2) MED, or 3) Green-MED diet. All subjects received free gym memberships and physical activity guidance. Both MED groups consumed 28 g walnuts/d (+440 mg/d polyphenols). The Green-MED group consumed green tea (3–4 cups/d) and Mankai (Wolffia-globosa strain, 100 g frozen cubes/d) green shake (+800 mg/d polyphenols).

**Results:**

Among 284 participants (88% men; mean age: 51 y; BMI: 31.2 kg/m^2^; *APOE‐*ε*4* genotype = 15.7%), 224 (79%) completed the trial with eligible whole-brain MRIs. The pallidum (−4.2%), third ventricle (+3.9%), and LVV (+2.2%) disclosed the largest volume changes. Compared with younger participants, atrophy was accelerated among those ≥50 y old (HOC change: −1.0% ± 1.4% compared with −0.06% ± 1.1%; 95% CI: 0.6%, 1.3%; P < 0.001; LVV change: 3.2% ± 4.5% compared with 1.3% ± 4.1%; 95% CI: −3.1%, −0.8%; P = 0.001). In subjects ≥ 50 y old, HOC decline and LVV expansion were attenuated in both MED groups, with the best outcomes among Green-MED diet participants, as compared with HDG (HOC: −0.8% ± 1.6% compared with −1.3% ± 1.4%; 95% CI: −1.5%, −0.02%; P = 0.042; LVV: 2.3% ± 4.7% compared with 4.3% ± 4.5%; 95% CI: 0.3%, 5.2%; P = 0.021). Similar patterns were observed among younger subjects. Improved insulin sensitivity over the trial was the parameter most strongly associated with brain atrophy attenuation (P < 0.05). Greater Mankai, green tea, and walnut intake and less red and processed meat were significantly and independently associated with reduced HOC decline (P < 0.05). Elevated urinary concentrations of the polyphenols urolithin-A (*r* = 0.24; *P* = 0.013) and tyrosol (*r* = 0.26; *P* = 0.007) were significantly associated with lower HOC decline.

**Conclusions:**

A Green-MED (high-polyphenol) diet, rich in Mankai, green tea, and walnuts and low in red/processed meat, is potentially neuroprotective for age-related brain atrophy.

This trial was registered at clinicaltrials.gov as NCT03020186.

## Introduction

Brain atrophy is age-related and can be measured with MRI as an early biomarker of cognitive impairment. Medial atrophy (e.g., hippocampal loss) might be a more sensitive marker of neurodegeneration and cognitive decline than standard hippocampal volume measurement (1), especially in early, preclinical stages (2).

Lifestyle and metabolic factors [e.g., high blood pressure (BP) and cholesterol] (3), metabolic components (e.g., obesity, insulin resistance) (4), and poor dietary habits (e.g., red and processed meat consumption) (5) are all associated with global and regional gray matter loss, cognitive decline, and Alzheimer disease (AD). Modifying these lifestyle factors might attenuate the risks of those outcomes (6).

The Mediterranean diet (MED) has previously been associated with lower rates of brain atrophy and AD (5). However, data on brain volume dynamics from MED intervention trials are sparse (7).

The beneficial association between MED and age-related neurodegeneration might be partially explained by the abundance of polyphenols in plant-based food sources (8), which have antioxidant and anti-inflammatory metabolites. Polyphenols can cross the blood–brain barrier (BBB) (9), reduce neuroinflammation (10), and induce cell proliferation and adult-onset neurogenesis in the hippocampus (11). A previous clinical trial demonstrated that MED further supplemented with extra virgin olive oil or walnuts, which are foods rich in polyphenols, might halt age-related cognitive decline (12); however, there is little intervention trial evidence of slowing age-related atrophy in response to polyphenol consumption, specifically in healthy middle-aged participants (13, 14).

As part of the 18-mo DIRECT PLUS (Dietary Intervention Randomized Controlled Trial Polyphenols Unprocessed Study), we examined the effects of a caloric-restricted MED and the “Green-MED” diet further enriched with high-polyphenol foods—green tea [mostly epigallocatechin gallate (EGCG)] (15) and Mankai (which contains ∼200 polyphenol compounds) (16)—and low in red and processed meat, combined with physical activity (PA) accommodation, on brain atrophy. We hypothesized that the MED diet groups, and specifically the Green-MED diet group, which doubled the polyphenol content provided in the diet, would exhibit attenuated brain atrophy compared with the healthy dietary guidelines (HDG) group.

## Methods

### Research design

DIRECT-PLUS (NCT03020186) was launched in May 2017 and conducted in a workplace which is in a remote area in Israel (Nuclear Research Center Negev, Dimona, Israel). Workers do not leave the workplace during the workday, and a monitored lunch is provided. Most clinical and medical measurements (anthropometric measurements, blood drawing) and lifestyle-intervention sessions were performed at the workplace's medical department. Among 378 volunteers, 294 met age (≥30 y of age) and abdominal obesity inclusion criteria [waist circumference (WC): men >102 cm, women >88 cm] or had dyslipidemia [triglycerides (TGs) >150 mg/dL; HDL cholesterol ≤40 mg/dL for men, ≤50 mg/dL for women]. Exclusion criteria were inability to perform PA; serum creatinine ≥2 mg/dL; serum alanine aminotransferase or aspartate aminotransferase >3 times above the upper normal limit; major illness that might require hospitalization; pregnancy or lactation; active cancer or chemotherapy treatment in the last 3 y; warfarin treatment; pacemaker or platinum implantation; or participation in a different trial.

The Soroka Medical Center Medical Ethics Board and Institutional Review Board provided ethics approval. All participants provided written consent and received no financial compensation.

### Randomization and intervention

All participants completed the baseline measurements and were randomly assigned in a 1:1:1 ratio via a computer-based program, stratified by sex and work status (to ensure equal workplace-related lifestyle features between groups), into 1 of the 3 intervention groups: HDG as an active control group, MED, or Green-MED. The 18-mo-long interventions were contemporaneous, and participants were not blinded to group assignment (open-label protocol). All lifestyle interventions were held in person, by intervention groups, and included 90-min nutritional and PA sessions. These sessions were performed by a multidisciplinary guidance team (physicians, clinical dietitians, and fitness instructors). Sessions were weekly during the first month, once a month during the subsequent 6 mo, and every other month thereafter. In addition, a website listing all nutritional and PA information needed by the participants to continue with the intervention was accessible to the participants according to their intervention group.

Furthermore, all participants received free gym membership. Further instructions, according to the intervention group, were as follows.

HDG group: in addition to PA, participants received standard nutritional counseling to promote a healthy diet and achieve a similar intervention intensity.

MED group: in addition to PA, participants were instructed to follow a calorie-restricted, traditional MED low in simple carbohydrates, as described in our previous CENTRAL (17) trial. The MED was rich in vegetables, with poultry and fish partly replacing beef and lamb. The diet included 28 g walnuts/d containing 440 mg polyphenols/d [gallic acid equivalents (GAE)], including mainly ellagitannins, ellagic acid, and its derivatives (18).

Green-MED group: in addition to PA, the MED group instructions, and walnuts, the Green-MED diet participants were instructed to avoid processed and red meat and consume more plant-based food. Participants were also instructed to drink 3–4 cups/d of green tea and 100 g of *Wolffia globosa* (Mankai) frozen plant cubes as a green shake dinner substitute. Mankai, a specific strain of *Wolffia globosa*, is an aquatic plant, considered a natural food source or “vegetable meatball” in Asian cuisines (19). Nutritionally, Mankai is rich in polyphenolic compounds (20) and protein and provides bioavailable essential amino acids (21), iron (22), and vitamin B-12. Together, they provided an additional daily intake of 800 mg polyphenols (GAE) beyond the polyphenol content in the prescribed MED (18). The MED and Green-MED diets were similarly calorie-restricted (1500–1800 kcal/d for men and 1200–1400 kcal/d for women). The MED diet components of the 2 intervention groups were identical. A detailed description of the intervention outline and polyphenols provided is available in **Supplemental Table 1** and **Supplemental Methods 1**, respectively. The walnuts, green tea, and Mankai were provided free of charge and distributed at the on-site clinic.

Assessment of nutritional intake and lifestyle habits was self-reported online using validated FFQs (23, 24), including green tea, walnuts, and *Wolffia globosa* intake evaluation. Adherence to the PA program was quantified using an electronic self-reported validated PA questionnaire (25). PA intensity levels were subsequently measured using metabolic equivalent for task (MET) units per week; each unit is defined as the ratio of work metabolic rate to the standard resting metabolic rate, and MET levels can range from 0.9 METs (sleeping) to 18 METs (fast running) (26). The questionnaires were administered at baseline, after 6 mo, and at the end of the trial. The closed workplace enabled monitoring the freely provided lunch and the intense dietary and PA sessions, which were provided at the same time and intensity to all 3 groups. Executive functions were measured using the Brief Executive Function (BEF) battery (27) at baseline and after 18 mo of intervention. Participants performed the BEF battery individually in an isolated, quiet room with a research assistant present only during the practice phase. The BEF is composed of 2 measurements. *1*) Working memory/decision load was evaluated by comparing performance in choice reaction-time (CRT) tasks (involving letters, digits, shapes) of 6 choices (6CRT) and 2 choices (2CRT). Specifically, we computed the Balanced Integration Score (BIS) (28) for both the 6CRT and 2CRT, and then predicted 6CRT from 2CRT using simple linear regression; the score was the residual from this regression. *2*) Switching was evaluated by comparing a task-switching condition (Switch), involving trial-to-trial shifts in the 2CRT tasks (e.g., shift from letter to digit to shape). This task was initially scored using the BIS. The final score was the residual from the regression in which Switch-BIS was predicted by 2CRT-BIS. Inhibition was assessed with the accuracy in the antisaccade task. Finally, mental speed was assessed by mean response time in the 2CRT.

**Supplemental Methods 2** details further assessment tool descriptions including brain MRI, a detailed BEF description (including **Supplemental Figures 1–3**), and methodology analysis.

### Statistical analysis

**FIGURE 1:**
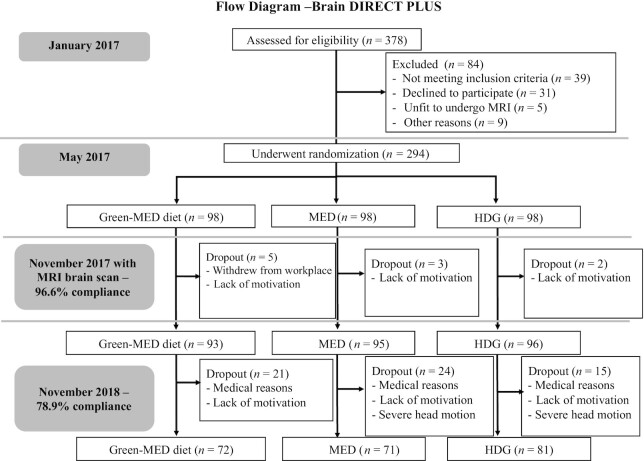
Flowchart of Brain DIRECT PLUS (Dietary Intervention Randomized Controlled Trial Polyphenols Unprocessed Study). Green-MED diet, Mediterranean diet higher in polyphenols and lower in red/processed meat; HDG, healthy dietary guidelines; MED, Mediterranean diet.

**Supplemental Methods 3** details the sample size calculations. The DIRECT PLUS primary outcomes were 18-mo changes in visceral abdominal tissue, intrahepatic fat, and adiposity. The results for the primary outcomes are presented in a separate report (29). Longitudinal data from DIRECT PLUS allowed us to examine the effects of the dietary interventions on brain volume and structures. **Figure 1** presents the complete flowchart of the Brain DIRECT-PLUS trial. Our a priori primary assessment was differences in changes in brain volumes: hippocampal occupancy score (HOC), calculated as hippocampal volume/[hippocampal volume + inferior lateral ventricle volume (LVV)] for each hemisphere, and then averaged between them (1), and the LVV. Our secondary endpoints were changes in white-matter tract integrity [diffusion tensor imaging (DTI) as measured by fractional anisotropy (FA)] and changes in executive function (EF; as a substudy). We also examined the degree to which changes in volume of brain structures were associated with clinical parameters, blood biomarkers, urine polyphenols, intake of specific food items, and changes in DTI and EF.

Continuous variables are presented as mean ± SD. Dependent variables were analyzed to determine whether they were normally distributed using the Kolmogorov–Smirnov test and visually inspected by histogram. Changes over time across 18 mo of intervention in anthropometric measurements, blood biomarkers, and MRI parameters within groups were measured using Wilcoxon tests or paired-sample *t* tests. Between-group differences were tested using independent-sample *t* tests, ANOVA, Mann–Whitney *U* tests, Kruskal–Wallis tests, or chi-square tests, depending on the number of groups and distribution of the dependent variables. Kendall τ correlation was used to examine the *P*-trend. Longitudinal changes in brain volumes are presented as the percentage of change relative to baseline. Changes in FA and EF over time are presented as absolute differences. Spline regression was performed to explore the optimal threshold for change in HOC across age after exploring each 5-y age interval. For urine polyphenol untargeted analysis, values of 0 were imputed to the lowest value in our sample. We performed log2 transformation to calculate the changes from baseline and assess the correlations between HOC and phenolic compounds. Changes in brain structures and associations with urinary phenolic compounds were corrected using the false discovery rate (0.05) method for multiple comparisons. Pearson or Spearman correlation was used to analyze associations of anthropometric measurement and blood biomarker changes with changes in brain volume structures. HOC and LVV observations of >2.5 SDs from the overall mean were deemed outliers and excluded from the analyses (5 HOC, 3 LVV). General linear models were computed to analyze the effects of the MED and Green-MED diets compared with HDG on HOC and LVV changes, and all the comparisons were further adjusted for potential confounders: age, sex, and insulin resistance (HOMA-IR), the parameter most strongly associated with brain atrophy attenuation. Multiple comparisons were addressed using Bonferroni correction. Statistical significance was set at *P* < 0.05. Statistical analyses were performed using SPSS version 25.0 (IBM Corp.). Graphs were created using GraphPad Prism version 8.2.1 (GraphPad Inc.).

## Results

### Baseline characteristics

**Table 1** presents baseline characteristics among the 284 participants with MRI brain scans. The mean participant age was 51 ± 10 y (median: 50 y; range: 31–82 y), and 88% were men. The mean BMI and WC were 31.2 ± 3.9 kg/m^2^ and 109.7 ± 9.5 cm, respectively. Baseline HOC, LVV, and hippocampal volume were similar across intervention groups (*P* > 0.05 for all). The *APOE‐*ε*4* genotype (≥1 allele) was equally distributed (*n* = 43, 15.2% heterozygotes) across intervention groups. Only 1 participant was homozygotic; overall, 15.7% had the *APOE‐*ε*4* variant.

**TABLE 1:** Baseline characteristics by intervention group (for the entire population and for participants aged ≥50 y at baseline)^1^

	Entire cohort (*n* = 284)					≥50 y of age (*n* = 139)				
	Entire	HDG (*n* = 96)	MED (*n* = 95)	Green-MED diet (*n* = 93)	Test statistic, *P* value	Entire	HDG (*n* = 51)	MED (*n* = 48)	Green-MED diet (*n* = 40)	Test statistic, *P* value
Anthropometric parameters										
Age (median)	51.1 ± 10.6 (49.9)	51.2 ± 10.6	51.5 ± 10.5	50.4 ± 10.9	0.9, 0.65	60.0 ± 7 (58.9)	59.6 ± 6.0	59.8 ± 7.4	60.8 ± 7.7	0.4, 0.8
Sex, % male of study population	88.4	87.5	88.4	89.2	0.1, 0.93	88.5	86.3	87.5	92.5	0.9, 0.63
BMI, kg/m^2^	31.2 ± 3.9	31.3 ± 3.8	31.3 ± 3.9	31.1 ± 3.9	0.4, 0.81	30.9 ± 3.4	31.1 ± 3.2	31.3 ± 3.7	30.1 ± 3.2	3.1, 0.21
Weight, kg	93.6 ± 14.4	92.8 ± 14.8	94.8 ± 13.5	93.3 ± 15.0	1.9, 0.37	90.9 ± 12.1	90.4 ± 11.8	93.3 ± 11.7	88.5 ± 12.7	2.8, 0.25
Waist circumference, cm	109.7 ± 9.5	109.9 ± 10.3	110.1 ± 9.6	109.0 ± 8.7	1.2, 0.54	109.1 ± 7.6	109.3 ± 8.6	110.0 ± 6.92	107.8 ± 6.8	2.8, 0.24
Systolic BP, mm Hg	130.4 ± 14.1	130.3 ± 14.4	130.4 ± 12.3	130.6 ± 15.4	0.02, 0.98	134.1 ± 14.8	133.6 ± 14.6	133.5 ± 12.7	135.4 ± 17.6	0.2, 0.81
Diastolic BP, mm Hg	81.1 ± 10.3	80.2 ± 11.4	81.9 ± 8.9	81.4 ± 10.6	0.7, 0.51	81.4 ± 10.8	79.9 ± 11.9	82.8 ± 9.4	81.5 ± 10.8	0.9, 0.42
Blood biomarkers										
Glucose, mg/dL	102.1 ± 17.3	102.1 ± 17.9	100.6 ± 13.4	103.5 ± 20.1	0.4, 0.82	106.1 ± 18.3	106.5 ± 20.1	104.1 ± 15.1	108.0 ± 19.4	0.6, 0.74
HOMA-IR	3.7 ± 2.3	4.0 ± 2.8	3.7 ± 1.9	3.6 ± 2.1	0.4, 0.81	3.7 ± 2.2	4.1 ± 2.7	3.6 ± 2.0	3.3 ± 1.8	2.1, 0.34
Total cholesterol, mg/dL	190.6 ± 33.4	192.6 ± 36.1	194.3 ± 31.7	184.7 ± 31.6	2.2, 0.11	190.1 ± 36.1	191.3 ± 41.1	191.0 ± 34.6	187.3 ± 31.6	0.2, 0.85
HDL-C, mg/dL	46.0 ± 11.7	45.6 ± 11.4	46.9 ± 11.0	45.5 ± 12.6	1.9, 0.39	48.3 ± 12.5	48.3 ± 12.2	48.8 ± 11.9	47.6 ± 13.8	0.9, 0.62
LDL-C, mg/dL	125.7 ± 31.1	127.4 ± 32.4	127.2 ± 31.4	122.5 ± 29.5	0.7, 0.47	124.1 ± 33.6	125.4 ± 36.2	123.4 ± 33.8	123.3 ± 30.6	0.1, 0.94
Triglycerides, mg/dL	145.6 ± 64.7	151.4 ± 69.2	152.7 ± 66.3	132.2 ± 56.0	4.4, 0.11	141.1 ± 61.0	141.3 ± 62.6	146.9 ± 67.6	133.6 ± 50.2	0.3, 0.86
*APOE*-ε*4* allele, %	15.7	18.1	11.7	17.4	1.7, 0.42	15.8	15.7	16.7	15.0	0.05, 0.98
MRI-derived brain anatomical parameters										
Hippocampal occupancy score	0.86 ± 0.1	0.86 ± 0.1	0.86 ± 0.1	0.86 ± 0.1	0.3, 0.88	0.83 ± 0.1	0.84 ± 0.1	0.84 ± 0.1	0.82 ± 0.1	0.4, 0.82
Lateral ventricle volume, cm^3^	25.3 ± 13.5	24.4 ± 11.8	24.6 ± 12.0	27.0 ± 16.2	0.7, 0.71	30.0 ± 15.9	29.3 ± 13.1	28.8 ± 12.9	32.5 ± 21.5	0.1, 0.97
Hippocampus volume, cm^3^	8.3 ± 0.9	8.2 ± 0.81	8.4 ± 0.8	8.4 ± 1.0	0.8,0.47	8.0 ± 0.9	8.0 ± 0.7	8.1 ± 0.9	7.8 ± 0.9	0.8, 0.45

1 Values are mean ± SD for continuous variables and *n* (%) for categorical variables, unless otherwise stated. *P* value according to ANOVA/Kruskal–Wallis test for continuous variables and chi-square test for categorical variables. A total of 284 participants had an available brain MRI scan at baseline. *ApoE*-ε*4* was considered positive if there was 1 *APOE*-ε*4* allele (only 1 participant had 2 alleles). BP, blood pressure; Green-MED diet, Mediterranean diet higher in polyphenols and lower in red/processed meat; HDG, healthy dietary guidelines; HDL-C, HDL cholesterol; LDL-C, LDL cholesterol; MED, Mediterranean diet.

**FIGURE 2:**
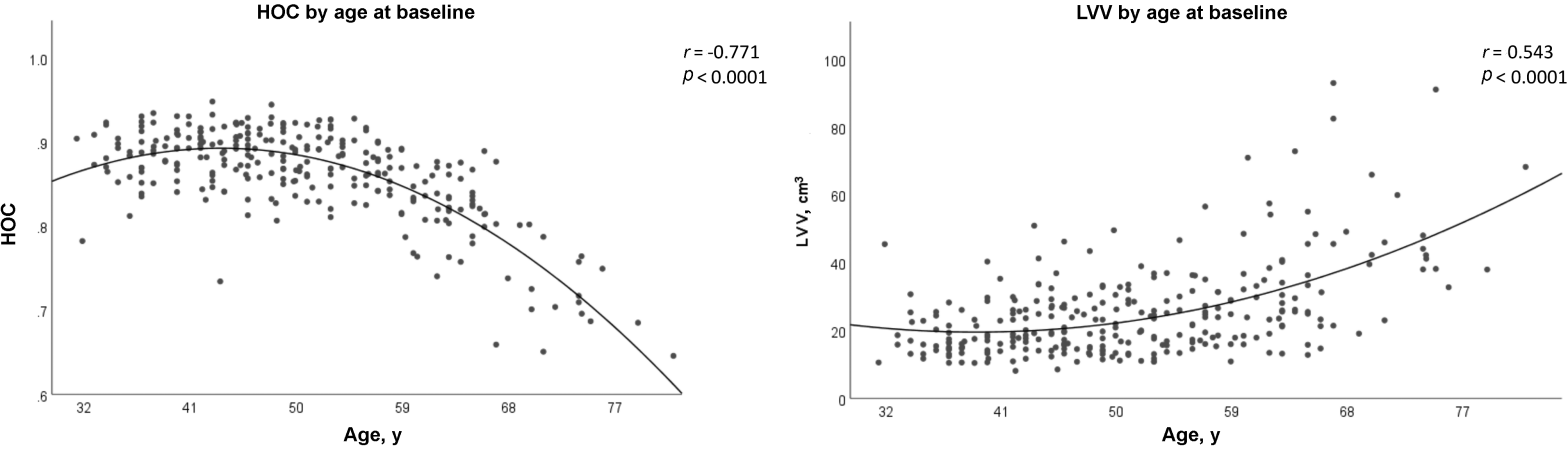
LVV and HOC across age at baseline. *n* = 284. Brain MRI-derived data were quantified and segmented in a fully automated manner using NeuroQuant. A change in slope was significant at age 50 y (*P*-nonlinear trend < 0.0001 for both, analyzed by spline regression). HOC, hippocampal occupancy score; LVV, lateral ventricle volume.

At the baseline phase, lower HOC (*r* = −0.77) and higher LVV (*r* = 0.54) were strongly correlated with age in a nonlinear fashion (*P* < 0.001) (**Figure 2**). After carefully exploring each 5-y age interval, age 50 y emerged as the most prominent threshold for changes in the slopes of HOC decline and LVV expansion (*t* = −9.86, *P* < 0.001). At baseline, aging was negatively correlated in a more linear pattern with FA in 5 out of the 6 investigated white-matter tracts: cingulum (*r* = −0.51), hippocampal cingulum (*r* = −0.32), uncinate fasciculus (*r* = −0.46), inferior longitudinal fasciculus (*r* = −0.17), and superior longitudinal fasciculus (SLF; *r* = −0.15) (*P <* 0.05 for all). Fornix FA was not associated with age (*r* = 0.06, *P* = 0.3). In the EF substudy among 200 participants at baseline, age was further significantly correlated with reduced EF parameters: mental speed (*r* = 0.56), inhibition (*r* = 0.46), working memory (*r* = −0.23), and task switching (*r* = 0.2) (*P <* 0.05 for all).

Higher HOC at baseline was significantly correlated with higher FA values in 4 out of 6 white-matter tracts and better conditions of all EF parameters. **Supplemental Figure 4** presents a full description of all baseline associations of anatomical brain structures, anatomical connectivity tracts, and EF parameters. After adjusting for age, only the uncinate fasciculus and SLF remained significantly correlated with HOC (*r* = 0.39, *P* < 0.001 and *r* = 0.12, *P* = 0.042, respectively), without any association between HOC and EF parameters. Higher HOC was correlated with lower WC (*r* = −0.17), lower systolic BP (*r* = −0.36), and lower diastolic BP (*r* = −0.13) (*P <* 0.05 for all). **Supplemental Table 2** presents the baseline associations of anthropometric measurements and blood biomarkers with HOC.

### Adherence to the lifestyle intervention and attrition rate

Among 284 participants with a brain scan at baseline, 224 (79%) completed the intervention and provided 2 valid brain scans. Dropout reasons were confined to a lack of motivation and medical reasons unrelated to the study. The attrition rate did not differ between intervention groups (HDG = 16%, MED = 25%, and Green-MED = 23%, *P* = 0.24 between groups). Noncompleters did not significantly differ from completers in baseline HOC (*P* = 0.57), age (*P* = 0.26), and sex (*P* = 0.36). In the EF substudy, 200 participants performed baseline measurements, and out of them, 120 participants had eligible 18-mo follow-up testing. As for the adherence to the lifestyle intervention, as previously reported (29) both MED groups had similar daily walnut intake amounts (*P* = 0.42) and were higher than the HDG group (*P* < 0.001 for HDG compared with both MED groups). The Green-MED diet was distinguished by higher green tea and Mankai green shake intake, along with reduced red meat and poultry intake, compared with the MED (*P <* 0.05 for all comparisons between MED groups). All participants significantly increased their PA (median: 28.4 MET units/wk postintervention compared with 23.2 at baseline, *P* < 0.001), and similarly across intervention groups (*P* = 0.28 between intervention groups). **Supplemental Results 1** and **Supplemental Table 3** provide further information regarding dietary intervention adherence.

### Age-dependent brain changes

**FIGURE 3:**
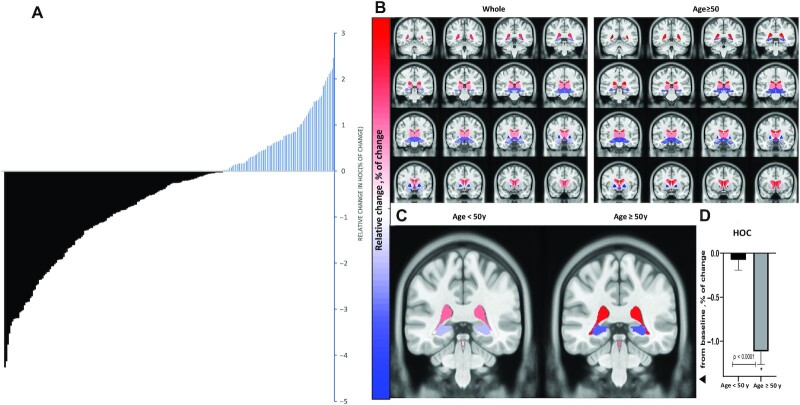
Dynamics in volume of different brain structures after 18 mo of lifestyle intervention [DIRECT-PLUS (Dietary Intervention Randomized Controlled Trial Polyphenols Unprocessed Study), *n* = 224]. (A) Individual response of change in HOC (% of change). A decrease in HOC is represented by black lines, an increase in HOC by blue lines. Among all participants who completed the study with 2 valid MRI brain scans, 148 (66%) exhibited a decrease in HOC after 18 mo. (B) Unbiased standard MRI template brain volume for the normal population, the distance of 4 slices or 4 mm between coronal cross-sections. The color represents the relative change (%) in volume of all available brain structures across the entire cohort and in participants ≥ 50 y of age. The analysis was corrected for multiple comparisons using the false discovery rate (0.05) method. (C) Changes in lateral ventricle volume (red) and hippocampal volume (blue) after 18 mo. Hippocampal atrophy and lateral ventricle expansion were significantly more prominent in older participants than in younger participants (*P* < 0.001 for both). The slice location is *y* = 96 in ab unbiased standard MRI template. (D) The relative decrease in HOC was significantly more prominent in older participants than in younger participants (cutoff = age 50 y, *P* < 0.001, independent-sample *t* tests). HOC, hippocampal occupancy score.

We first examined the overall change in brain volume after 18 mo and found that 66% of the participants exhibited a decline in HOC (**Figure 3**A). Figure 3B and **Supplemental Table 4** present brain volume and structure data. Overall, hippocampal volume decreased by 1.7% in the 18-mo interval. The most dynamic brain structures were the pallidum (−4.2%), third ventricle (+3.9%), and LVV (+2.2%). The most stable structure was the brainstem (0%). Among participants aged ≥50 y (*n* = 113), hippocampal volume decreased by 2.3%, pallidum decreased by 4.8%, and LVV increased by 3.2% (*P <* 0.05 compared with baseline for all).

Among those ≥50 y of age, 78.8% exhibited HOC decline. Decreases in HOC and LVV expansion were most apparent among participants ≥ 50 y old compared with younger participants (mean HOC change: −1.0% ± 1.4% compared with −0.06% ± 1.1%; 95% CI: 0.6%, 1.3%; *P* < 0.001; mean LVV change = 3.2% ± 4.5% compared with 1.3% ± 4.1%; 95% CI: −3.1%, −0.8%; *P* = 0.001) (Figure 3C, D).

### Effects of the intervention on longitudinal brain structure volume changes

**FIGURE 4:**
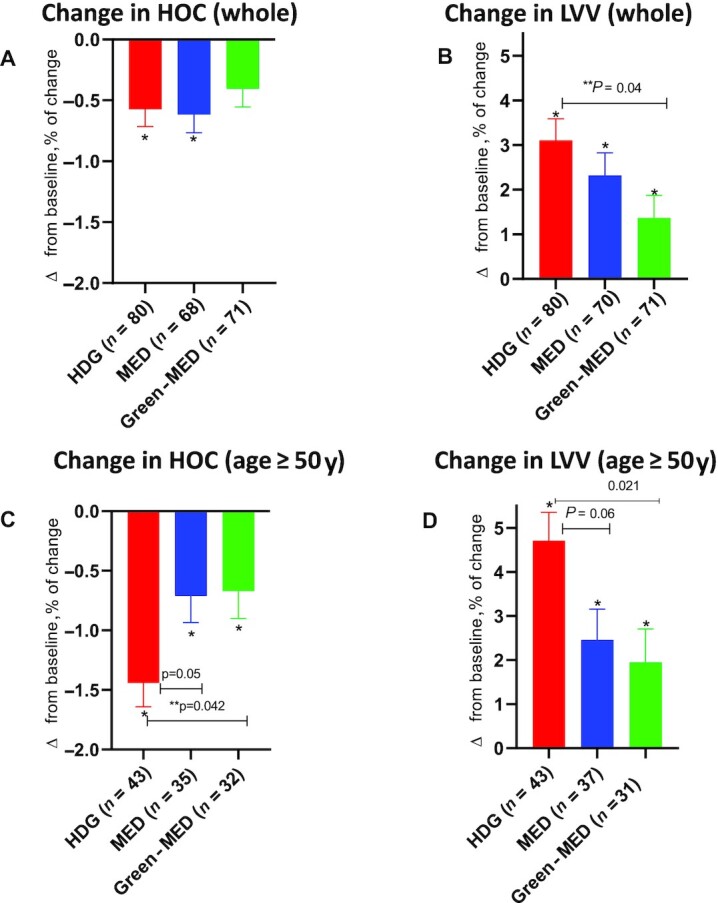
The effect of 18-mo dietary interventions on MRI-derived brain volume structures. (A) No significant difference in change in HOC (from baseline) between groups of the entire study population. The HDG and the MED group participants had a significant decrease in HOC. In contrast, participants in the Green-MED group did not have a significant change in HOC after 18 mo of intervention. (B) Compared with the HDG group, the Green-MED group had attenuated LVV expansion across the entire study population. (C, D) Among participants ≥ 50 y of age, both the MED and Green-MED diet groups demonstrated less HOC decline and LVV expansion than the HDG group. The analysis was performed with general linear modeling. Comparisons of changes in HOC and LVV between intervention groups were analyzed using Bonferroni correction for 3 comparisons. The age-by-group interaction was significant for change in HOC (*P*-interaction = 0.017) and marginal for change in LVV (*P*-interaction = 0.07). The time-by-group interaction in change in HOC was significant among participants ≥ 50 y old (*P*-interaction = 0.032). *Significant change from baseline within groups. **Significant difference between groups. Green-MED diet, Mediterranean diet higher in polyphenols and lower in red/processed meat; HOC, hippocampal occupancy score; LVV, lateral ventricle volume; MED, Mediterranean diet.

Owing to a statistically significant age-by-group interaction (age 50 y as threshold, *P* = 0.018), the following analyses were performed separately for those aged ≥ 50 y, as well as for the entire study group (**Figure 4**).

Among participants >50 y old, those assigned to either of the MED diets had a significantly lower decline in HOC (−0.78% ± 1.34% compared with −1.3% ± 1.4%; 95% CI: −1.26%, −0.24%; *P* = 0.005) and a smaller increase in LVV (2.4% ± 4.4% compared with 4.3% ± 4.5%; 95% CI: 0.84%, 4.1%; *P* = 0.003) than HDG participants. Specifically, the Green-MED diet group had a lower decline in HOC than the HDG-assigned participants (−0.8% ± 1.6% compared with −1.3% ± 1.4%; 95% CI: −1.52%, −0.02%; *P* = 0.042). Participants in the MED group had a marginally lower decline in HOC than the HDG group (−0.8% ± 1.2% compared with −1.3% ± 1.4%; 95% CI: −0.001%, −1.5%; *P* = 0.05). LVV expansion was also attenuated in Green-MED dieters compared with the HDG group (2.3% ± 4.7% compared with 4.3% ± 4.5%; 95% CI: 0.3%, 5.2%; *P* = 0.021). There was also a marginal beneficial effect of MED compared with HDG (2.7% ± 4.2% compared with 4.3% ± 4.5%; 95% CI: 0.07%, 4.6%; *P* = 0.06).

In the entire age cohort, LVV expansion was significantly attenuated in the Green-MED group compared with the HDG group (1.2% ± 4.1% compared with 3.1% ± 4.6%; 95% CI: 0.06%, 3.4%; *P* = 0.04), whereas no other significant differences were detected across groups. Neither white-matter tracts nor EF parameters were differently affected by the interventions.

### Association between changes in clinical parameters and brain volume dynamics

There were no significant interactions between *APOE‐*ε*4* genotypes and brain volume dynamics (*P* > 0.05 for HOC and LVV change). **Table 2** presents the associations of changes in anthropometric measurements and metabolic biomarkers with changes in HOC and LVV. For participants ≥ 50 y of age, weight loss, lower HOMA-IR, and lower TG concentrations were associated with a lower decline in HOC (*P <* 0.05 for all). Multivariate analyses indicated that only a favorable change in HOMA-IR (β: −0.26; 95% CI: −0.4, −0.04; *P* = 0.012) was independently associated with a favorable change in HOC (adjusted for age, sex, change in weight, and change in TG concentrations). Weight, BMI, and WC loss, as well as HOMA-IR and BP decline, were associated with a favorable change of LVV (*P <* 0.05 for all) (Table 2). With regard to white-matter tract measures, only total cholesterol (*r* = −0.18, *P* = 0.008) and LDL cholesterol decline (*r* = −0.14, *P* = 0.041) were associated with a favorable change of FA in the SLF (data not shown). No other associations were observed between changes in white-matter tracts or EF and clinical parameters.

**TABLE 2:** Associations of 18-mo MRI-derived changes in HOC and LVV with changes in anthropometric measurements and blood biomarkers^1^

	18-mo relative change in HOC				18-mo relative change in LVV			
	Entire		Age ≥ 50 y		Entire		Age ≥ 50 y	
	*r*	*P* value	*r*	*P* value	*r*	*P* value	*r*	*P* value
∆Weight	0.008	0.904	−0.211	0.027	0.233	<0.001	0.411	<0.001
∆BMI	0.011	0.875	−0.21	0.028	0.226	0.001	0.392	<0.001
∆Waist circumference	0.02	0.769	−0.11	0.254	0.213	0.001	0.246	0.009
∆Systolic BP	−0.145	0.034	−0.175	0.069	0.139	0.041	0.134	0.195
∆Diastolic BP	−0.142	0.038	−0.171	0.075	0.232	0.001	0.182	0.056
∆HOMA-IR	−0.111	0.108	−0.301	0.002	0.147	0.031	0.231	0.015
∆Triglycerides	−0.011	0.869	−0.206	0.032	0.086	0.202	0.084	0.381
∆Cholesterol	−0.017	0.799	−0.052	0.590	−0.035	0.606	−0.088	0.360
∆HDL-C	−0.037	0.592	0.015	0.878	−0.087	0.198	−0.116	0.225
∆LDL-C	−0.01	0.879	0.024	0.808	−0.03	0.658	−0.104	0.277

1 Univariate correlation matrix of 18-mo relative change in MRI HOC and LVV and 18-mo changes in selected parameters of the entire cohort and in participants who were ≥50 y of age at baseline. Brain MRI-derived data were quantified and segmented in a fully automated manner using NeuroQuant. The analysis was performed using Pearson or Spearman correlation, depending on the variable distribution. BP, blood pressure; HDL-C, HDL cholesterol; HOC, hippocampal occupancy score; LDL-C, LDL cholesterol; LVV, lateral ventricle volume.

### Association of consumption of specific dietary components and changes in urinary polyphenol biomarkers with the change in HOC

**FIGURE 5:**
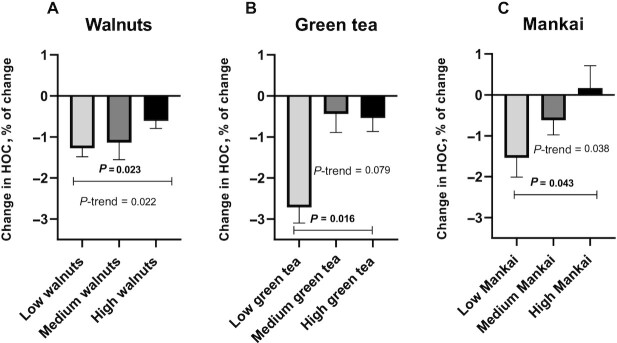
Relative change in HOC according to specific “green” dietary components (age ≥ 50 y). (A) Weekly walnut consumption: low was defined as ≤2/wk (*n* = 28), medium as 3–4/wk (*n* = 33), and high as ≥5/wk (*n* = 47). (B) Daily green tea consumption: low was defined as ≤1/d (*n* = 4), medium as 2/d (*n* = 6), and high as ≥3/d (*n* = 22). (C) Mankai consumption: low was defined as <3/wk (*n* = 12), medium as 4–6/wk (*n* = 13), and high as daily (*n* = 7). All analyses were performed for data from older participants of the study population (age ≥ 50 y). Mankai and green tea consumption were measured and analyzed only for the Green-MED diet (Mediterranean diet higher in polyphenols and lower in red/processed meat) group. All analyses were nonparametric. HOC, hippocampal occupancy score.

We explored the association of consumption of selected high-polyphenol provided components (walnuts, green tea, and Mankai) with changes in HOC scores. **Figure 5** presents the results for those ≥50 y of age who most benefited from the intervention. Greater Mankai consumption (Green-MED dieters) was associated with attenuated HOC decline (dose-response, *P*-trend = 0.038, *P* = 0.043 between the highest and lowest consumption groups). Increased green tea consumption was marginally associated in the same direction (*P*-trend = 0.079, *P* = 0.016 between the highest and lowest consumption groups). Across groups, increased walnut consumption was also associated with a lower decline in HOC (dose-response, *P*-trend = 0.022, *P* = 0.023 between the highest and lowest consumption groups). Furthermore, reduced red and processed meat consumption was associated with an attenuated decline in HOC compared with no change in consumption (*P* = 0.047 and *P* = 0.042, respectively) (**Supplemental Figure 5**). Among the urine polyphenol biomarkers, elevated concentrations of urine urolithin A (median: 5.5; IQR: 4.8–6; *r* = 0.17, *P* = 0.012) and tyrosol (median: 5.7; IQR: 5.5–5.8; *r* = 0.14, *P* = 0.036) were significantly associated with a lower decline in HOC in the entire sample and in subjects ≥ 50 y of age (median: 5.6; IQR: 4.9–6.1; *r* = 0.24, *P* = 0.013, and median: 5.7; IQR: 5.6–5.8; *r* = 0.26, *P* = 0.007 for urolithin A and tyrosol, respectively).

## Discussion

This 18-mo MRI PA + dietary clinical trial among 284 participants with abdominal obesity suggests that the Green-MED, high-polyphenol diet is potentially neuroprotective against age-related neurodegeneration. Overall, we observed a significant increase in hippocampal atrophy, which was accelerated in participants over age 50 y compared with younger participants. Consumption of specific “green” dietary components such as Mankai, green tea (marginally), and walnuts and reduced consumption of red meat were specifically associated with reduced HOC decline, as were increases in specific urine Mankai-derived polyphenols (i.e., urolithin A and tyrosol). Moreover, improvements in cardiometabolic parameters such as glycemic markers, body weight, and BP were associated with less HOC decline and LVV expansion. Our findings underscore the beneficial role of a healthy lifestyle that includes consumption of dietary polyphenols, particularly on brain health in early aging.

The hippocampus is an essential subcortical region for learning and memory (30). Accumulated evidence suggests that hippocampal atrophy is a key part of the mechanism contributing to the development of age-related neurodegenerative diseases. HOC is a measure of hippocampal atrophy and has been suggested to be more sensitive and predictive than standard volumetric hippocampal measurement for cognitive decline (1). In our trial, the baseline correlation with age was stronger with HOC than with standard hippocampal volume, and there were significant baseline correlations of HOC with FA values and EF parameters.

We also found a robust nonlinear correlation between age and brain volumes at baseline (e.g., HOC and LVV) and a significant slope change at age 50 y, as well as a significant age-by-intervention group interaction after 18 mo. Our findings are in line with previous studies showing that the trajectories of hippocampal volume and LVV vary nonlinearly by age (31, 32) and that hippocampal atrophy accelerates around age 55 y (33). Our findings also corroborate previous evidence that white-matter integrity tends to deteriorate with age and that processing (or mental) speed, a core component of cognitive abilities, is one of the earliest cognitive abilities to decline (34).

Although *APOE*‐ε*4* genotype is a well-known genetic risk factor for AD, we did not find an association of *APOE*‐ε*4* status with brain volumes at baseline or with trajectories of brain volumes after 18 mo of lifestyle intervention. This might be explained by the low prevalence of the *APOE*‐ε*4* genotype in our study population and the uncertain effect of the *APOE*‐ε*4* genotype on brain atrophy in healthy participants (35).

Our study provides support for the beneficial effects of lifestyle interventions on brain health in older adults. We showed that improved glycemic control, weight loss, and decreased BP were correlated with attenuation of brain atrophy. Sedentary lifestyle, obesity, high BP, and lower glycemic control have been established as substantial risk factors for brain atrophy and cognitive decline (4, 36), and higher cardiovascular disease risk scores at baseline may predict faster rates of cognitive decline and brain atrophy (36). Along these lines, a previous interventional study demonstrated that 1 y of aerobic exercise selectively increased hippocampal volume by 2% (37). Moreover, an intensive lifestyle intervention to induce weight loss was associated with lower LVV in patients with type 2 diabetes mellitus (14). In addition, regional brain atrophy can manifest years before an age-related neurodegenerative disease is diagnosed (1), highlighting the importance of intensive lifestyle interventions in early adulthood to control modifiable risk factors.

Our main finding shows that the MED and Green-MED diets had more potent effects on HOC and LVV atrophy among older adults than the control condition of HDG. These results are in concordance with prior evidence, demonstrating that lower adherence to the MED was associated with age-related brain atrophy in the elderly after 3 y regardless of age, sex, and other cardiovascular disease risk factors (38). Moreover, less meat consumption was associated with lower brain atrophy rates. This finding is in accordance with a cross-sectional study (5) that found an inverse association between brain volumes and meat consumption.

A previous large interventional study of 334 older participants demonstrated a beneficial effect of a MED (augmented by polyphenols in the form of olive oil or walnuts) on cognitive function (specifically in memory and learning) over 4 y of intervention (12). However, other studies failed to show an improvement in cognitive function and/or beneficial effects on brain anatomy after a MED intervention. Similarly, in our trial and in contrast to the volumetric findings, we could not detect between-group differences, which was perhaps due to a relatively short intervention time, small sample size on the EF substudy, and lack of sensitivity of the methods used to assess cognitive decline (7). Furthermore, we did not find an intervention effect on white-matter integrity. The evidence that dietary pattern affects white-matter connectivity is relatively weak, but it has been suggested that MED adherence is associated with higher FA values. White-matter integrity might mediate the relation between dietary pattern and cognitive function (39, 40). This discrepancy might be partially accounted for by the different nature of our trial (as a longitudinal intervention (and the robust harmful effect of aging on white-matter integrity (41).

In our trial, both MED groups received 28 g walnuts/d, which may protect against age-related cognitive decline, mild cognitive impairment, and AD by reducing inflammation and oxidative stress (42). Although we found a significant association between increased walnut consumption and attenuation of brain atrophy (as measured by HOC), we failed to demonstrate a significant direct effect of 18 mo of dietary intervention on cognition. This finding is in concordance with a previous long-term intervention trial (43) that did not show a significant effect on cognition after 2 y of walnut supplementation. This might be explained by factors related to the experimental design, namely, insufficient intervention duration, the small sample size in the EF substudy, and the relatively young and healthy study population.

Participants in the Green-MED group were instructed to consume 3–4 cups/d of green tea containing polyphenols (mostly EGCG), which has been associated with improved cognitive function, reduced neuroinflammation, amyloid β aggregation, microglial activation, and neurodegenerative diseases, both in vivo and in vitro (15). Participants also consumed a Mankai green shake daily, which contained flavonoids (e.g., kaempferol quercetin, catechin) reported to enhance cognition and memory and delay the appearance of neurodegeneration markers (15) and to be inversely related to the incidence of dementia and cognitive decline (44). In the present study, increased consumption of Mankai green shake and green tea (marginally) were associated with attenuated HOC decline. This finding might be partly explained by the beneficial effect of Mankai on the glycemic response, which was an essential mediator of attenuating brain atrophy in our previous trial (45). Notably, previous interventional studies that examined the effect of polyphenols on MRI-measured brain atrophy did not always reveal a beneficial effect on brain volume (13). The effect found in our study might be accounted for by the long and intensive intervention, a larger sample size, and augmentation of the dietary intervention with PA, which has been shown to improve brain volume (37).

Polyphenols from plant-based foods are potent antioxidants that can cross the BBB (9) and ameliorate neuroinflammation by inhibiting proinflammatory markers such as IFN-γ, IL-6, NF-κB, and TNF-α (10, 46). Polyphenols can also increase cerebral blood flow, induce hippocampal neurogenesis and plasticity (11, 47), improve white-matter integrity and spatial learning, and increase locomotor activity in mice (48) and humans (47). Among the urine polyphenols, urolithin A and tyrosol elevations were associated with favorable brain anatomic changes. Urolithin A is a gut metabolite of ellagic acid (49), which is found in Mankai and walnuts, and a higher level of consumption was correlated with increased urolithin A concentrations. Urolithin A can cross the BBB, inhibit neuroinflammation, promote neurogenesis, and specifically protect the hippocampus from oxidative stress (50). Tyrosol, which is found predominantly in olive oil, was also shown to protect the hippocampus from neuroinflammation, stimulate neurogenesis, and improve spatial memory (51).

Several limitations of this study should be noted. First, there was no passive or placebo control group as a reference, and all groups received PA accommodation, including free gym memberships, which might have had an additive effect on brain atrophy attenuation. This might have led to underestimating the effect of the dietary interventions. However, we decided not to include a passive control group in our trial design for ethical and motivational reasons. In addition, because this was not a drug, food supplement, or food extract trial but a dietary pattern trial with specific high-polyphenol foods provided as a case study, we cannot claim an effect of specific foods or nutrients. Urine polyphenol measurements are limited in reflecting polyphenol intake and vary widely in their specificity and sensitivity across different polyphenol compounds (52). However, our study did show a correlation between increments in specific polyphenol secretion, which are proposed to reduce neuroinflammation (49), and brain atrophy. Most of our participants were men, reflecting the workplace population. This limits our ability to generalize our results to women, although age-related brain atrophy is more pronounced in males and starts earlier (53).

Moreover, data regarding educational status, baseline cognitive impairment, and depression were not available, so we could not stratify or exclude these conditions in our analysis. Finally, our study focused on brain anatomy and included cognitive functions only as a substudy. However, medial temporal atrophy (i.e., lower HOC) was associated with decreased white-matter integrity and lower EF capabilities at baseline in prior research (1). The strengths of this study include a relatively large sample size for the primary outcome of this trial; long duration; integrated data of brain volumetric analyses, white-matter integrity, and EF capabilities; and high adherence. In addition, the median age of the study participants was 50 y, an optimal age for lifestyle intervention because hippocampal atrophy accelerates at this age (54) and begins years before apparent clinical manifestations of cognitive decline (2). Furthermore, brain volume measured by MRI at baseline and postintervention and quantifying brain volume by a validated automated tool might improve the sensitivity of the analysis in detecting brain volume loss compared to the traditional manual radiologist approach (55). Another strength is the closed workplace environment from which participants were recruited, which enabled monitoring of the provided lunch, the presence of an on-site clinic at the participants’ workplace, intense dietary guidance, group meetings with multidisciplinary guidance, and access to polyphenol-rich food sources provided free of charge.

In conclusion, a calorie-restricted Green-MED diet enriched with specific polyphenols and decreased red and processed meat consumption may amplify the beneficial effects of a traditional calorie-restricted MED on age-related brain atrophy in participants mainly >50 y of age, together with PA accommodation. The results from this study might suggest a simple, safe, and promising avenue to slow age-related neurodegeneration. Future studies are needed to explore the exact mechanisms of specific polyphenol-rich foods on brain anatomy and function.

## Supplementary Material

nqac001_Supplemental_File

## Data Availability

Data described in the article, code book, and analytic code will be made available upon request pending approval of the principal investigator (irish@bgu.ac.il).

## Notes

Supported by German Research Foundation (DFG) project number 209933838 (SFB 1052; B11) (to I Shai); Israel Ministry of Health grant 87472511 (to I Shai); Israel Ministry of Science and Technology grant 3-13604 (to I Shai); and the California Walnut Commission (to I Shai). AK is a recipient of the Kreitman Doctoral Fellowship at the Ben-Gurion University of the Negev. None of the funding providers were involved in any stage of the design, conduct, or analysis of the study, and they had no access to the study results before publication.

Supplemental Tables 1–4 , Supplemental Methods 1–3, Supplemental Figures 1–5, and Supplemental Results 1 are available from the “Supplementary data” link in the online posting of the article and from the same link in the online table of contents at https://academic.oup.com/ajcn/.

Abbreviations used: AD, Alzheimer disease; BBB, blood–brain barrier; BEF, Brief Executive Function; BIS, Balanced Integration Score; BP, blood pressure; CRT, choice reaction-time; DTI, diffusion tensor imaging; EF, executive function; EGCG, epigallocatechin gallate; FA, fractional anisotropy; GAE, gallic acid equivalents; Green-MED diet, Mediterranean diet higher in polyphenols and lower in red/processed meat; HDG, healthy dietary guidelines; HOC, hippocampal occupancy score; LVV, lateral ventricle volume; MED, Mediterranean diet; MET, metabolic equivalent for task; PA, physical activity; SLF, superior longitudinal fasciculus; TG, triglyceride; WC, waist circumference.
