# Supplementary material for: The effect of a high-polyphenol Mediterranean diet (Green-MED) combined with physical activity on age-related brain atrophy: the Dietary Intervention Randomized Controlled Trial Polyphenols Unprocessed Study (DIRECT PLUS)
Source: Am J Clin Nutr. 2022 Jan 11;115(5):1270–81. doi: 10.1093/ajcn/nqac001 (PMC9071484; doi:10.1093/ajcn/nqac001)
Supplement: nqac001_Supplemental_File [file nqac001_supplemental_file.docx]

**Online-Only Supplemental Material**

**The effect of a high-polyphenol Mediterranean diet (GREEN-MED) combined with physical activity on age-related brain atrophy: the DIRECT PLUS randomized controlled trial Running title: The effect of Green-MED diet on brain atrophy**

Alon Kaplan MD ^1^, Hila Zelicha MPH^1^, Anat Yaskolka Meir PhD^1^, Ehud Rinott MMedSc^1^, Gal Tsaban MD^1,2^, Gidon Levakov MSc^3,4^, Ofer Prager PhD^4^, Moti Salti PhD^4^, Yoram Yovell MD-PhD ^5^, Jonathan Ofer MMedSc^4^, Sebastian Huhn PhD^6^, Frauke Beyer PhD^7^, Veronica Witte PhD^7^, Arno Villringer MD^7^, Nachshon Meiran PhD^3^, Tamar Bakun Emesh MA^3^, Peter Kovacs PhD^8^, Martin von Bergen PhD^9^, Uta Ceglarek PhD^10^, Matthias Blüher MD^11^, Michael Stumvoll MD^11^ ,Frank B Hu MD-PhD^12,13^, Meir J Stampfer MD, Dr.P.H ^12,13^ , Alon Friedman MD-PhD^4,14^, Ilan Shelef MD ^1,2^, Galia Avidan PhD^3,4^, Iris Shai PhD^*1,12^

| **Title** | **Page** |
| --- | --- |
| Supplementary methods 1: Provided polyphenol-rich products | 2-3 |
| Supplementary methods 2: Further laboratory methodology, brain anatomy and executive function assessment | 3-9 |
| Supplementary methods 3: Sample size calculation | 9 |
| Supplementary results 1: Additional data of adherence to lifestyle intervention | 10 |
| Supplementary Table 1 – outline of the lifestyle intervention | 11 |
| Supplementary Table 2: Associations between MRI derived Hippocampal occupancy score and selected parameters at baseline | 12 |
| Supplementary Table 3: 18-month changes in reported dietary intake across intervention | 13-14 |
| Supplementary Table 4: Changes in brain structures volume after 18 months | 15-16 |
| Supplementary figure 1 - The Choice Reaction Time Task | 17 |
| Supplementary figure 2 - The Switching Task | 18 |
| Supplementary figure 3 - The Anti-saccade Task | 19 |
| Supplementary Figure 4: Baseline associations of MRI derived brain volumes, anatomical connectivity tracts, and executive function at baseline | 20 |
| Supplementary Figure 5: Change in hippocampal occupancy score by meat consumption | 21 |
| References | 22-24 |

**Online-Only Supplemental Material – table of content**

**Supplementary Methods**

**Supplementary methods 1: Provided polyphenol-rich products**

Walnuts [groups Mediterranean (MED), green-MED]: The main polyphenols in walnuts are ellagitannins, ellagic acid, and its derivative(1). Walnuts are considered to have a beneficial effect on health maintenance and disease prevention(2). Besides, walnuts consumption is associated with decreased risk for dementia as well as reducing neuroinflammation(3).

Green tea (green-MED): an unfermented tea produced from the leaves of Camellia sinensis, is prepared by drying and steaming the leaves, and is a rich source of polyphenols(4). Most of the polyphenols found in green tea are Catechins (the monomer form of flavanols), mainly epigallocatechin (EGC), epicatechin gallate (ECG), and epigallocatechin gallate (EGCG)(5). These compounds were associated with improved cognitive function and reduced neuroinflammation(6,7).

Wolffia globosa duckweed - Mankai (group green-MED): A specific strain of Wolffia globosa an aquatic plant, which can serve as a plant protein source. In Asian cuisines, Wolffia globosa is considered a natural food source or "vegetable meatball"(8). Nutritionally, Mankai is characterized by high protein content (more than 45% of the dry matter), and the presence of 9 essential and 6 conditional amino acids(9). The Mankai plant is rich in polyphenols compounds(10) and provides bioavailable essential amino acids(9), iron(11), Vitamin B12(12) and beneficial effects on postprandial and fasting glycemic control(13). We guided the participants to prepare a green Mankai shake with additional ingredients, which were also part of the diet regimen (fruits, walnuts, or vegetables) each evening. The green protein shake partially substituted for dinner, replacing beef/poultry protein sources. The green-MED pattern was also recently suggested to further decrease cardiovascular risk of compared to MED diet(14).

**Supplementary methods 2: Further laboratory methodology, brain anatomy and executive function assessment**

Clinical parameters and blood biomarkers

All parameters were measured at baseline and after 18 months of intervention. Waist circumference was measured to the nearest millimeter halfway between the last rib and the iliac crest using an anthropometric measuring tape. Resting blood pressure (BP) was measured twice using an automatic blood pressure monitor (Accutorr 4, Datascope); the average between the measurements was computed. Blood and urine samples were collected at 8:00 am after a 12-hour fast. Blood samples were centrifuged and stored at -80°C. Serum total cholesterol (TC; Coefficient-of-variation (CV), 1.3%), HDL-c, low-density-lipoprotein-cholesterol (LDL-c), and triglycerides (CV, 2.1%) were determined enzymatically with a Cobas 8000 automatic analyzer (Roche). Plasma glucose levels were measured by Roche GLUC3 (hexokinase method). Plasma insulin levels were measured with an chemiluminesence immunometric assay (CLIA) from Diasorin (; CV, 2.5%). Homeostasis model assessment for insulin resistance (HOMA−IR) was calculated using the following equation: HOMA−IR=Fasting insulin (μU/mL) x Fasting glucose (mg/dL)/405.(15) The apolipoprotein E ε4 allele genotype, a main susceptibility gene variation for Alzheimer's disease (AD),(16) was determined by genotyping rs429358 and rs7412 single nucleotide polymorphisms (SNPs) using TaqMan™ SNP Genotyping assay technology according to the manufacturer’s protocol. The genotype calls were generated by using the endpoint measurement module from the Applied Biosystems™ 7500 Real-Time PCR System (Applied Biosystems, Inc., Forster, CA). The corresponding APOE genotypes were defined based on the two variants as described elsewhere.(17) To limit genotyping errors, a random 5% selection of samples was regenotyped; all genotypes matched initially designated genotypes.

Brain magnetic resonance imaging and quantitative volumetric analyses

Brain structure volumes were assessed at baseline and 18 months using the 3D T1-TFE sequence with 1.0 mm isometric voxels using an inversion pulse sequence. The imaging parameters were TR=2500 msec and TE=30 ms, which were determined with a 240x220x150 matrix field of view. The acquisitions were carried out using a 3.0-T Philips Ingenia. An MR technologist reviewed scans for quality at the time of acquisition.

Brain structure volumes were quantified using NeuroQuant®(18,19) at both time points. The NeuroQuant computer-automated analysis routinely provides volume data on 15 brain regions in both hemispheres for a total of 30 volume measurements (http://www.cortechs.net/products/neuroquant.php). This procedure provides fully automated segmentations involving several steps, including stripping the skull and mapping the brain with a Talairach atlas. The automatic segmentation process includes image filtering, artifact correction, segmentation, error measurement, and report generation. NeuroQuant has been validated and approved by the Food and Drug Administration as a tool for providing quantitative segmental volumes. All T1 MRIs were deidentified before uploading to the NeuroQuant software. Based on previous data, our primary outcome was the hippocampal occupancy score (HOC). The HOC is a measure of ex vacuo dilation, indicating expansion of the temporal horn of the lateral ventricle at the expense of the hippocampal volume. HOC might help to identify hippocampal shrinkage resulting from brain atrophy as opposed to a congenitally small hippocampus.(18) Our secondary outcome was lateral ventricle volume (LVV), a measure of AD progression, and the rate of LVV change is correlated with an increase in senile plaques.(20)

Diffusion tensor imaging analysis:

Diffusion-weighted imaging was acquired using echo-planar imaging with the following parameters (TR=4364 msec, TE= 80 ms, 1.75 mm in-plane resolution, 112x112x64 acquisition matrix, 128x128x64 reconstruction matrix, 2mm slice thickness, 2 mm gap between slices, axial slice orientation, flip angle=90°, diffusion-weighting: B= 1000 mm²/s, 32 diffusion directions). Due to a scanner upgrade in March 2017, there was a change in total EPI readout time from 0.053s to 0.0415s.
Preprocessing was performed using a publicly available nipype pipeline in python 2.7. (<https://github.com/fBeyer89/DIRECT_PLUS>) which included software tools from MRTrix 3.0, FSL 6.0.1 and AFNI 19.1.05. DWI images were first denoised using dwidenoise and mrdegibbs. Then, eddy-cuda was used to simultaneously correct susceptibility artifacts, eddy currents, inter- and intra-volume subject movement and signal dropout. Finally, we used DTIfit to fit the diffusion-tensor to the eddy-corrected data.
FA images from participants who had DTI data at both timepoints (N=219) were processed with a longitudinal implementation of FSL’s tract-based spatial statistics (TBSS)(21). We co-registered baseline and 18 months FA images into a common space, applied TBSS to the average and then applied the skeletonization to the co-registered FA maps from both time points.
We used the ICBM WM atlas to extract average, skeletonized FA values for 6 WM tracts (Cingulum, Hippocampal Cingulum, Fornix, inferior/superior longitudinal fasciculus and uncinate fasciculus) which have been previously been associated with modifiable risk factors and lifestyle factors(22).
Quality control was performed using [eddyqc](https://fsl.fmrib.ox.ac.uk/fsl/fslwiki/eddyqc/UsersGuide) and by visual inspection and none of the participants had to be excluded due to head motion or other artifacts.

Executive function analysis:

**(**This section was adapted and slightly modified from a previous publication of our group(23))

EF were assessed using the BEF battery including tests measuring Switching, Inhibition and Working-Memory (WM)/decision-load functioning. The BEF battery consists of:

Choice Reaction Time (CRT).

This test began with three 6-choice-reaction tasks (high WM load/decision complexity, 6CRT) with tasks involving letters, digits and shapes, respectively (see Fig. S1A, bottom). Each task comprised 72 trials preceded by 6 trials for practice. In all three tasks, the mapping between stimuli and response keys (on the keyboard) was arbitrary and thus required keeping this mapping information in WM. Participants used the index, middle and ring fingers of their right and left hands to respond in this task. In the next phase, three additional 2-choice-reaction tasks (low WM load/decision-complexity, 2CRT) were executed, with tasks involving letters, digits and shapes, respectively (Fig. S1A, top) and comprising 36 trials each, preceded by 2 trials of practice. Participants used just the index fingers of both hands, with the two choices mapped to the same stimuli as in the previous (6-choice) phase. Thus, WM-load was reduced in the 2-choice condition by both having fewer rules to keep in mind (two vs. six) and by the fact that these rules were trained beforehand. All the task-stimuli were presented at the center of a black 19-in. (48.26-cm) computer screen. The stimuli for the CRT were the Hebrew letters “ת,ש,ר,ק,צ,פ,ס,נ,מ,ל,כ,ט,ח,ז,ד,ג,ב” (Hebrew was the language of the participants), digits 0–9 which were presented using 48-point Times New Roman font. The shapes were 8 symmetrical shapes (Fig. S1) printed in white against a black background. Each shape was 64 × 64 pixels in size. The task cues stimuli were “W” for letters, “%” for numbers and “○” for shapes. Each task cue was 64 × 64 pixels in size. Stimuli were presented in white on a black background. Each trial included a fixation (500 ms) followed by the target accompanied by a task cue which were presented until the response was given or until 6 sec had elapsed. Errors were followed by a 400 ms visual feedback (Fig. S1B).

Switching task.

This task was introduced after the CRT tasks and used the already learned 2-choice stimulus-response mapping rules with task cues that were already familiar. The task began with a screen displaying the task cues and the stimulus-response mapping (Fig. S2A) and continued with a sequence of trials in which the task (letter, digit, shape) switched in every trial (Fig S2B). Presentation of letters, shapes, and digits as well as error indication were the same as in the CRT task, with the only difference being that each target stimulus comprised of a combination of a shape, a letter and a digit (Fig. S2B). There were 201 trials in this task preceded by 6 practice trials. Two distracting effects were controlled for.

The anti-saccade task

This task measured inhibition success. This task comprising of 96 trials, each started with a centered fixation mark (+) that appeared for a variable duration (1,000 ms and 2,000 ms in 500 ms intervals). It was followed by a cue presented on one side of the screen (e.g., left) for 200–350 ms in 50 ms intervals. The cue was followed by the presentation of a target stimulus on the opposite side (e.g., right) for 100 ms before being masked by gray cross-hatching that disappeared after response or after 5 sec. The visual cue was a white square (64 × 64 pixels), and the target stimulus was a small white arrow (64 × 64 pixels). The participants’ task was to indicate the direction of the arrow (left, up, down or right) with the keyboard's arrows (Supplementary Materials Fig. S3). Participants were required to inhibit the reflexive response of looking at the initial cue (a small white square) because doing so would make it difficult to correctly identify the direction of the arrow. The task started with 24 practice trials.

Scoring

We generated four scores. Speed, was the average RT in the 2CRT. WM functioning/decision-complexity was operationalized by the discrepancy between performance in 6CRT (high WM load/decision-complexity) and the 2CRT (low load). High scores represent compromised WM efficiency and/or poor ability to make decisions under load. Switching was operationalized by the discrepancy between the switch condition and the 2-choice condition, where high scores representing difficulty in handling switching. For computing both WM functioning/decision under load and Switching, we used BIS scores of 2CRT, 6CRT, and Switch. To compute BIS, we used the following formula:

(1)

$${BIS}_{i,j}= Z_{{PC}_{i,j}}-Z_{\bar{{RT}_{i,j}}}$$

Residual scores were calculated by predicting participants' BIS in 6CRT from the 2CRT-BIS (*b_0_* and *b_1_* are optimal regression weights computed on the results of the normative sample. They of course have different values when predicting 6CRT-choice performance and when predicting switching performance):

(2)

$$\tilde{Performance}_{6-choice or switching}=b_{0}+b_{1}\left( {Performance}_{2-choice} \right)$$

(3)

$$Residual score= {Performance}_{6-choice or switching}-\tilde{Performance}_{6-choice or switching}$$

A similar procedure was used to compute residual scores in Switch (except that Switch replaced the 6CRT in Equations 2 and 3). Before actual computing of performance measures for all three effects, data were cleaned by removing trials quicker than 150 ms and slower than 3 SDs above participant's average for each condition. Before calculating RT, error and post-error trials were removed as well. This is how data are typically treated in such tasks.

The Z-scores (needed to compute BIS) and the regression formulae we both conducted on a national representative sample of 544 17 years-old Israel Defense Forces recruits. This choice was taken to ensure a stable anchor for the scores. Of course, given the age differences, the scores cannot be interpreted as reflecting norms, and can only be used to compare between conditions/groups.

Urine polyphenols metabolites:

Acetonitrile (ACN) and Water in LC-MS analytical grade were purchased from J.T. Baker (Part of Fisher Scientific). Formic acid was purchased from Honeywell (Charlotte, NC, USA), and β-glucuronidase (EC: 3.2.1.31) type HP-2 from H.pomatia was obtained from Sigma Aldrich (St.Louis, MO, USA).

Urine samples were thawed at RT for 5-10 min. For enzymatic deconjugation 15 µl β-glucuronidase were added to 50 µl urine and incubated for 2 h at 37 °C. Samples were then extracted twice by adding 600 µl ethyl acetate and shaking for 5 min at 1100 rounds per minute (RPM). The organic phases were combined and centrifuged (15 min, 3000 RPM) to remove impurities. Subsequently, the supernatant was and dried in a SpeedVac™ vacuum concentrator (Eppendorf) and stored.

Prior to LC-MS/MS measurement the samples were resuspended in water with 1% ACN and 0.1% formic acid and 10 µl were injected into an HPLC-QToF instrument from Agilent Technologies (6540 UHD Accurate-Mass Q-TOF LC/MS instrument) Metabolites were loaded on a C18-precolumn (Acquity BEH C18 1.8 µm, 2.1 x 50 mm) separated on a C18 column (Acquity UPLC HSS T3 1.8 µm, 2.1 x 100 mm) at a flow rate of 0.3 mL/min with the following gradient of running solvent A (0.1% formic acid in water) and running solvent B (0.1% formic acid in acetonitrile): 0-5 min 1% B, 5.1-20 min 1%-100% B, 20.1-25 min 1% B. All samples were acquired in positive and negative ionization mode. The QToF was set up in centroid mode and in screening mode allowing the detection of ions with a mass to charge ratio between 60 and 1000. After each full scan, the 5 most intense ions (threshold 200 counts) were fragmented.

Raw files (.d) were imported into the Progenesis QI® software (v.2.1, Waters Corporation). Samples in different ionization modes were processed separately. The workflow included isotope and adduct fusion and chromatogram alignment in tR direction based on a reference chromatogram was done. Next, peak picking was applied using default sensitivity settings. A database search was performed using ChemSpider as an identification method with the urine human metabolome database(24) and Phenol explorer(25) as input selection. Precursor and fragment mass tolerance were set to 15 ppm and 10 ppm, respectively. Only precursor peaks with a corresponding fragment spectra were kept. Normalized peak areas and possible identifications were exported.

The exported possible feature identifications were filtered using in-house written R scripts(26), Briefly, feature identifications were filtered for Progenesis score of at least 40. Then for each feature, only the top scoring identification and those which had a score less than 5 lower than the top score was kept. Finally, the resulting filtered data were further analyzed using a second in-house written R-script to extract possible phenolic compounds in the identification list. Only identifications where the phenolic compound was the top-scoring hit or shared top scoring hit was annotated as a phenolic compound.

**Supplementary methods 3:** **Sample size calculation**

The sample size calculation was based on the hippocampal gray matter volume. To detect a difference in hippocampal gray matter volume was based on a previous 1-year intervention study in which 120 participants were randomized into two groups (60 in each group): exercise and stretching(27). The detection means are 0.16 and 0.23 cm^3^ difference in the left and right hippocampus volume, respectively. Since neither the delta standard deviation nor the pooled variance is supplied, we created this estimation table. With a power of 80% and a significance level of 5%, the sample size calculations are as follows:

| Sample size for left hippocampus (in each group) | 25 | 50 | 74 | 99 | 123 |
| --- | --- | --- | --- | --- | --- |
| Sample size for right hippocampus (in each group) | 12 | 24 | 36 | 48 | 60 |
| Pooled variance | 0.04 | 0.08 | 0.12 | 0.16 | 0.20 |

We speculated that the pooled variance of the difference between groups would be 0.12 cm^3^. Thus, the required sample size of 74 subjects in each group.

**Supplementary results 1: Additional data of adherence to lifestyle intervention:**

Among the green-MED group, the 18-month daily green tea consumption weighted average (accounting for the reported consumption after six months and the reported consumption

after additional 12 months) was a median=3. IQR (1.5-4) cups per day. The median consumption of Mankai=3, IQR (1.5-4) shakes per weak.

| **Green-MED** | | **MED** | **HDG** |  |
| --- | --- | --- | --- | --- |
| 18-months group sessions in the workplace, weekly for the first month and monthly thereafter  18 months free gym membership  18 months of PA educational sessions  45-60 minutes of aerobic training + resistance training, 3-4 times/week | | | | Lifestyle group sessions, including PA |
| Limit dietary cholesterol, trans-fat, saturated fat, sugars, and salt and increase intake of vegetables | | | | General dietary guidance |
| 1500-1800 kcal/day for men, 1200-1400 kcal/day for women | | | Guidelines for a healthy MED diet with no specific recipes or calorie restriction | Energy, kcal/day |
| ~40% mainly PUFA and MUFA | | |  | Total fat, % of daily consumption |
| Less than 40 gr/day in the first 2 months with increased gradual intake for up to 80 gr/day | | |  | Carbohydrates, gr/day |
| Less/Avoid red and processed meats. Reduced poultry intake | | |  | Specific recommendations |
| +1240 mg/day  [source: provided walnuts (28 g/day), green tea (3-4 cups/day), Wolffia globosa duckweed (Mankai) shake (100 g frozen cubes)] | +440 mg/day  [source: provided  walnuts (28g/day] | |  | Polyphenols, mg/day |

**Supplementary Table 1 – outline of the lifestyle intervention**

**Supplementary Table 2: Associations between MRI derived Hippocampal occupancy score and selected parameters at baseline (DIRECT-PLUS n=284):**

| **Baseline associations with Hippocampal occupancy score** | | | | |
| --- | --- | --- | --- | --- |
| **Age**≥**50** | | **Entire** | |  |
| **p value** | **r** | **p-value** | **r** |  |
| **<0.001** | -**0.726** | **<0.001** | **-0.56** | Age (years) |
| 0.97 | -0.003 | 0.57 | 0.034 | Sex |
| 0.51 | 0.056 | 0.93 | -0.005 | Weight (Kg) |
| 0.22 | -0.105 | 0.13 | -0.09 | BMI (Kg/m^2^) |
| ***0.05*** | ***-0.17*** | **0.005** | **0.17-** | Waist circumference (cm) |
| **<0.001** | **-0.34** | **<0.001** | **-0.36** | Systolic BP (mm/Hg) |
| 0.82 | -0.02 | **0.034** | **-0.13** | Diastolic BP (mm/Hg) |
| **0.012** | **-0.212** | **<0.001** | **-0.267** | HbA1C (%) |
| 0.75 | 0.028 | 0.34 | -0.057 | HOMA IR |
| 0.24 | 0.1 | 0.31 | 0.061 | Triglycerides (mg/dL) |
| **0.001** | **0.283** | **0.011** | **0.151** | Cholesterol (mg/dL) |
| 0.28 | -0.092 | 0.11 | 0.095 | HDLc (mg/dL) |
| **0.001** | **0.271** | **0.034** | **0.126** | LDLc (mg/dL) |

**Supplementary table 2 legend:** Baseline associations of MRI Hippocampal occupancy score and selected parameters of the entire cohort and in participants ≥50 years of age. Brain MRI derived data was fully automated quantified and segmented using Neuroquant. BMI, body mass index; BP, blood pressure; HDLc, high density lipoprotein cholesterol; LDLc, low density lipoprotein cholesterol; HOMA-IR, homeostatic model assessment of insulin resistance. The analysis was made using Pearson or Spearman correlation, depending on the variables distribution.

**Supplementary Table 3: 18-month changes in reported dietary intake across intervention groups**

|  | **HDG (n=81)** | **MED (n=71)** | **Green-MED (n=72)** | **p between groups** | **P between MED groups** |
| --- | --- | --- | --- | --- | --- |
| **Red meat** | | | | | |
| More | 6.2 | 6.8 | 4.2 |  |  |
| Same | 51.9 | 45.2 | 26.4 | **0.013** | **0.032** |
| Less | 42 | 47.9 | 69.4 |  |  |
| **Processed meat** |  |  |  |  |  |
| More | - | - | - |  |  |
| Same | 37 | 35.6 | 29.2 | 0.56 | 0.48 |
| Less | 63 | 64.4 | 70.8 |  |  |
| **Legumes** |  |  |  |  |  |
| More | 23.5 | 26 | 40.3 |  |  |
| Same | 64.2 | 54.8 | 38.9 | **0.032** | 0.12 |
| Less | 12.3 | 19.2 | 20.8 |  |  |
| **Nuts** |  |  |  |  |  |
| More | 32.1 | 58.9 | 56.9 |  |  |
| Same | 63 | 31.5 | 27.8 | **<0.001** | 0.57 |
| Less | 3.9 | 15.3 | 15.3 |  |  |
| **Walnuts** |  |  |  |  |  |
| More | 42 | 78.1 | 81.9 |  |  |
| Same | 56.8 | 19.2 | 15.3 | **<0.001** | 0.83 |
| Less | 1.2 | 2.7 | 2.8 |  |  |
| **Green tea** |  |  |  |  |  |
| More | 30.9 | 38.4 | 80.6 |  |  |
| Same | 59.3 | 52.1 | 16.7 | **<0.001** | **<0.001** |
| Less | 9.9 | 9.6 | 2.8 |  |  |

**Supplementary table 3 legend:** data are presented in percentage. Data was extracted from the food frequency questioners that were self-reported by the participants. Difference in specific food consumption were analyzed using chi-square test.

**Supplementary Table 4: Changes in brain structures volume after 18 months**

| **Participants ≥50** **years of age** | | **All participants** | | |
| --- | --- | --- | --- | --- |
| **Q value** | **Change (percent of change** | **Q-value** | **Change (percent of change)** | **Brain structure** |
| 0.006 | -2.3 | 0.001 | -1.7 | Hippocampus |
| 0.01 | 3.2 | 0.003 | 2.2 | Lateral ventricles |
| 0.02 | 3.5 | 0.002 | 3.9 | Third ventricle |
| 0.006 | 2.0 | NS | 1.4 | Forth ventricle |
| NS | 0 | NS | 0 | Fifth ventricle |
| 0.003 | -2.7 | 0.001 | -2.2 | Ventral diencephalon |
| 0.01 | -4.8 | 0.002 | -4.2 | Pallidum |
| 0.002 | 2.5 | 0.006 | 1.5 | Nucleus accumbens |
| 0.002 | 1.6 | 0.004 | 1.5 | Thalamus |
| 0.002 | 2.3 | 0.005 | 1.5 | Caudate nucleus |
| NS | 0.07 | NS | -0.2 | Putamen |
| NS | -0.3 | 0.004 | -1.2 | Amygdala |
| NS | 2.0 | NS | 2.4 | Cerebellum |
| NS | -0.4 | NS | 0 | Brain stem |
| NS | 0.3 | NS | 0.4 | Forebrain |

**Supplementary table 4 legend:** Percent of change in all available brain structures after 18 months. Q-value were calculated using false discovery rate method and presented only for structures that remain statistically significant after correction for multiple testing.

**Supplementary figure 1 - The Choice Reaction Time Task.**

**
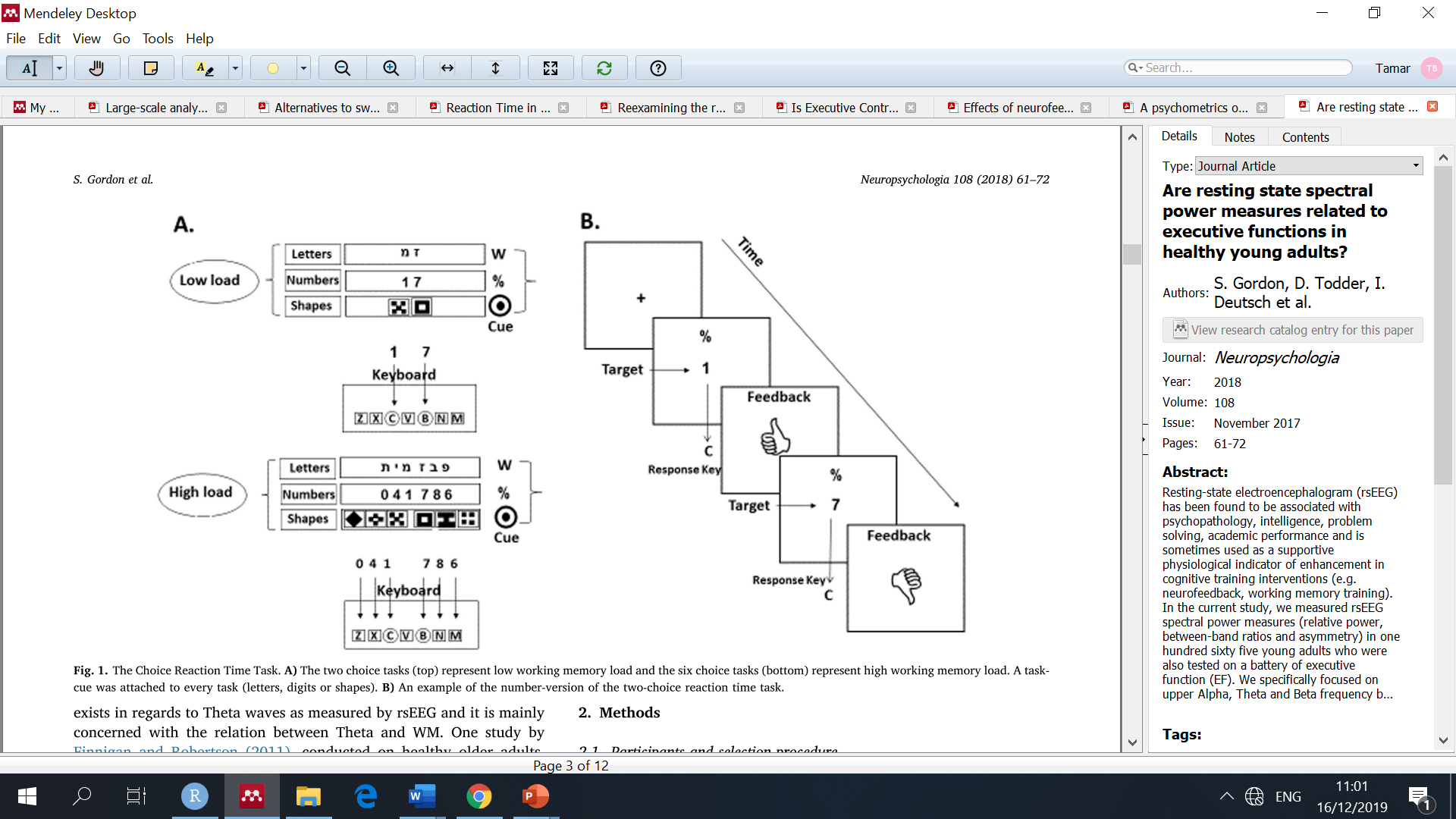
**

**Supplementary figure 1 legend:** The 2-choice tasks (top) represent low working memory/decision load and the 6-choice tasks (bottom) represent high working memory/decision load. A task-cue was attached to every task (letters, digits or shapes). B. An example of the number-version of the 2-choice reaction time task.

**Supplementary figure 2 - The Switching Task.**

**
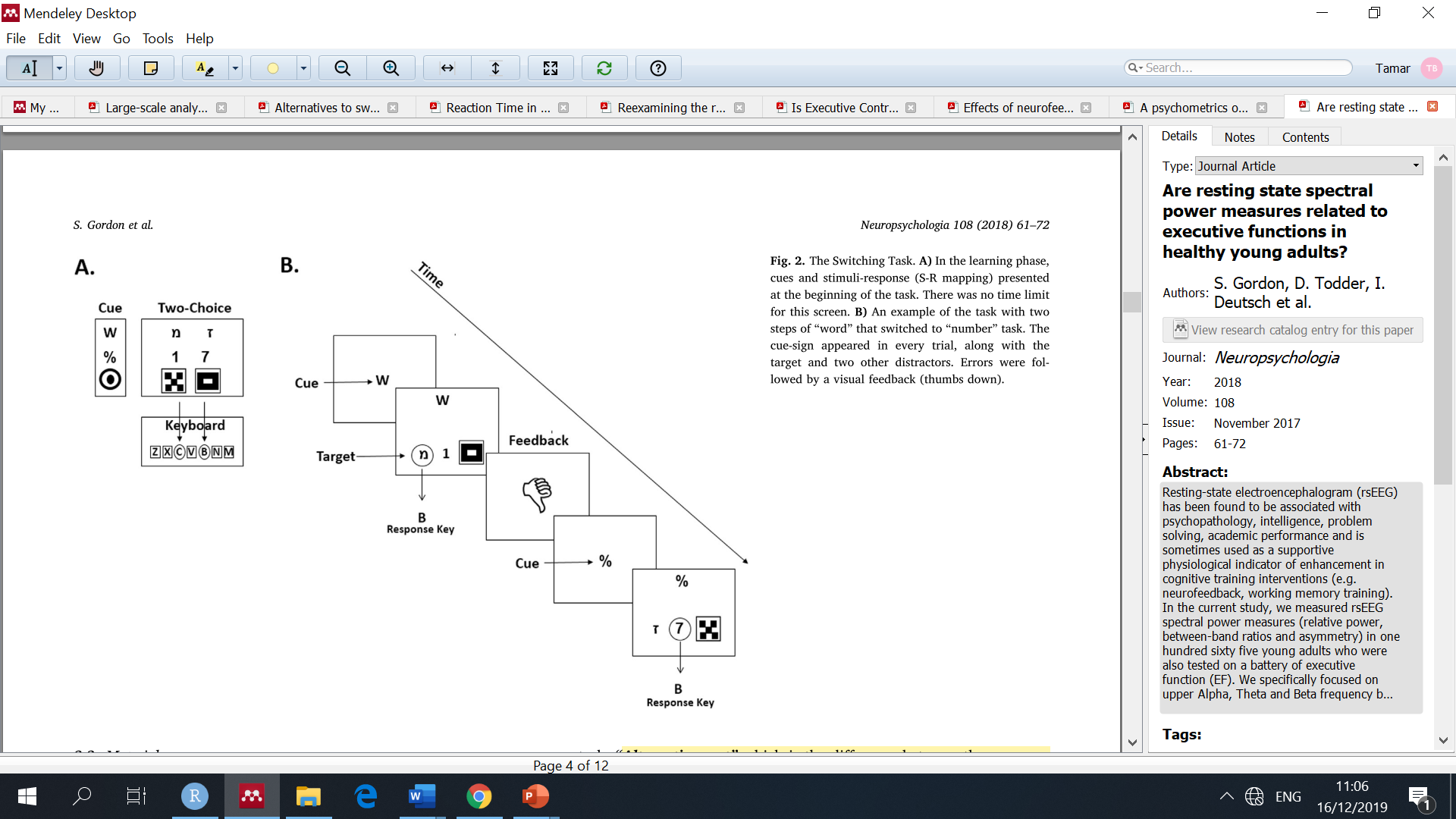
**

**Supplementary figure 2 legend**: A. In the learning phase, cues and stimuli-response (S-R mapping) were presented at the beginning of the task. There was no time limit for this screen. **B.** An example of the task with two steps of “word” that switched to “number” task. The cue-sign appeared in every trial, along with the target and two other distractors. Errors were followed by a visual feedback (thumbs down).

**Supplementary figure 3 - The Anti-saccade Task.**

**
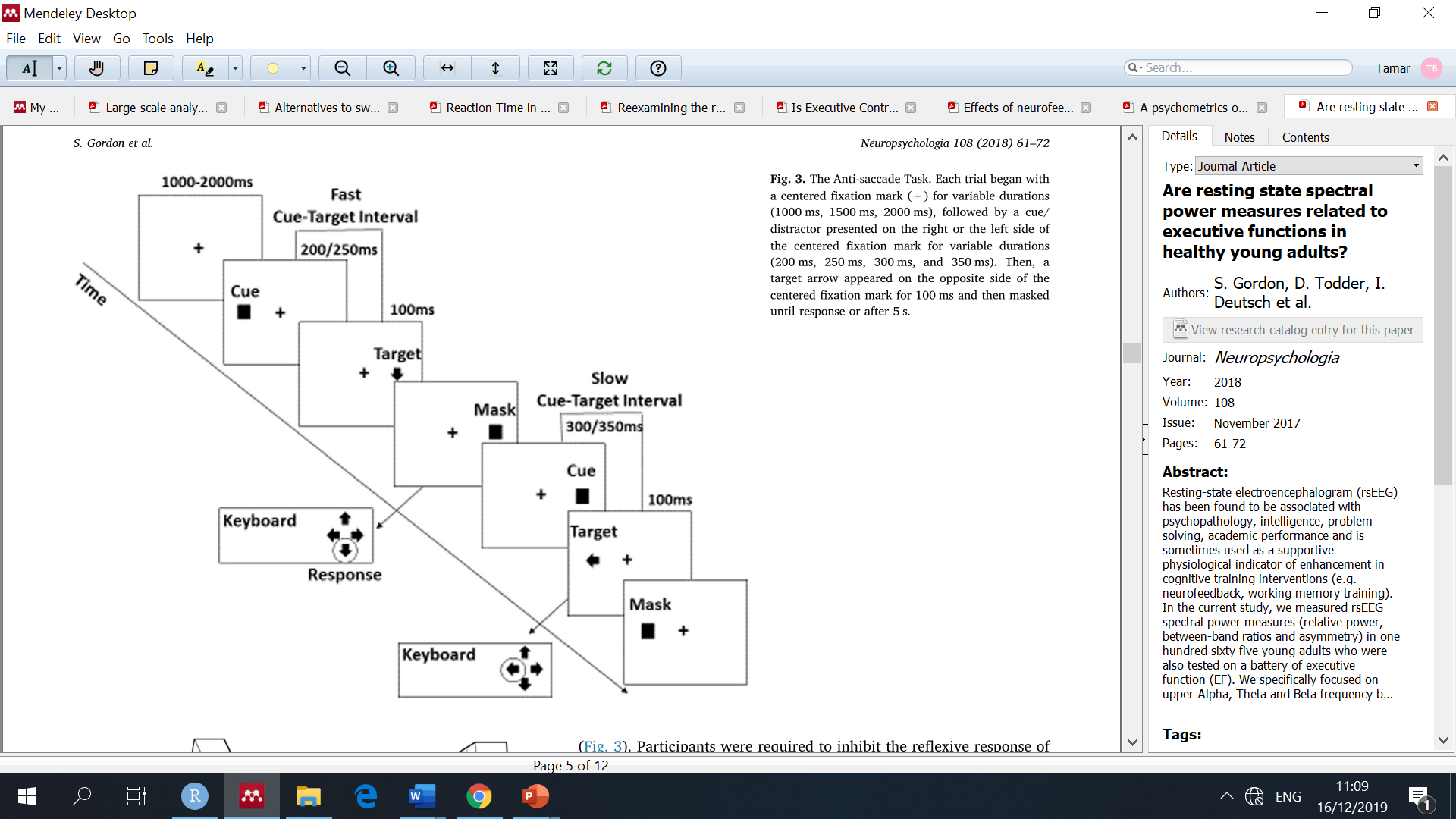
**

**Supplementary figure 3 legend:** Each trial began with a centered fixation mark (+) for variable durations (1000 ms, 1500 ms, 2000 ms), followed by a cue/ distractor presented on the right or the left side of the centered fixation mark for variable durations (200 ms, 250 ms, 300 ms, and 350 ms). Then, a target arrow appeared on the opposite side of the centered fixation mark for 100 ms and then masked until response or after 5 sec.

**Supplementary Figure 4: Baseline associations of MRI derived brain volumes, anatomical connectivity tracts, and executive function at baseline**

**Supplementary figure 4 legend:** Heat map of baseline correlations of all brain volumetric parameters with FA and EF parameters. HOC and LVV are presented in volume (cm^3^), the anatomical connectivity tracts are presented in fractional anisotropy (FA) values, mental speed is presented in milliseconds. Other executive function parameters are presented as described in the executive function methods. At the extremes of the color gradient, red represents the strongest positive correlation (r = +1), and green represents the strongest inverse correlation (r = -1); white or no color represents no correlation (r = 0). * denotes significant correlation at 0.05 level and ** denotes significant correlation at 0.01 level.

.

**Supplementary Figure 5: Change in hippocampal occupancy score by meat consumption**

**Supplementary figure 5 legend:** The relative change in HOC according to meat consumption. Tha analysis was performed with Mann–Whitney U test for data from older participants of the Green-MED group.

**References**

1. Regueiro J, Sánchez-gonzález C, Vallverdú-queralt A, Simal-gándara J, Lamuela-raventós R, Izquierdo-pulido M. Comprehensive identification of walnut polyphenols by liquid chromatography coupled to linear ion trap – Orbitrap mass spectrometry. Food Chemistry Elsevier Ltd; 2014;152:340–8.

2. Sánchez-gonzález C, Ciudad CJ, Noé V. Health benefits of walnut polyphenols : An exploration beyond their lipid profile. Critical Reviews in Food Science and Nutrition Taylor & Francis; 2017;57:3373–83.

3. Chauhan A, Chauhan V. Beneficial effects of walnuts on cognition and brain health. Nutrients 2020;12:1–10.

4. Pérez-Jiménez J, Neveu V, Vos F, Scalbert A. Identification of the 100 richest dietary sources of polyphenols: An application of the Phenol-Explorer database. European Journal of Clinical Nutrition 2010;64:S112–20.

5. Manach C, Williamson G, Morand C, Scalbert A, Rémésy C. Bioavailability and bioefficacy of polyphenols in humans. I. Review of 97 bioavailability studies. The American journal of clinical nutrition. 2005.

6. Xu Y, Zhang J, Xiong L, Zhang L, Sun D, Liu H. Green tea polyphenols inhibit cognitive impairment induced by chronic cerebral hypoperfusion via modulating oxidative stress. The Journal of nutritional biochemistry Elsevier Inc.; 2010;21:741–8.

7. Spencer JPE, Vafeiadou K, Williams RJ, Vauzour D. Neuroinflammation: Modulation by flavonoids and mechanisms of action. Molecular Aspects of Medicine Elsevier Ltd; 2012;33:83–97.

8. Bhanthumnavin K. Mcgarry MG. Wolffia globosa. Nature 1971;

9. Kaplan A, Zelicha H, Tsaban G, Yaskolka Meir A, Rinott E, Kovsan J, Novack L, Thiery J, Ceglarek U, Burkhardt R, et al. Protein bioavailability of Wolffia globosa duckweed, a novel aquatic plant – A randomized controlled trial. Clinical Nutrition [Internet] Elsevier Ltd; 2019;38:2576–82. Available from: https://doi.org/10.1016/j.clnu.2018.12.009

10. Daduang J, Vichitphan S, Daduang S, Hongsprabhas P, Boonsiri P. High phenolics and antioxidants of some tropical vegetables related to antibacterial and anticancer activities. African Journal of Pharmacy and Pharmacology 2011;5:608–15.

11. Yaskolka Meir A, Tsaban G, Zelicha H, Rinott E, Kaplan A, Youngster I, Rudich A, Shelef I, Tirosh A, Brikner D, et al. A Green-Mediterranean Diet, Supplemented with Mankai Duckweed, Preserves Iron-Homeostasis in Humans and Is Efficient in Reversal of Anemia in Rats. Journal of Nutrition Oxford University Press; 2019;149:1004–11.

12. Sela I, Meir AY, Brandis A, Krajmalnik-Brown R, Zeibich L, Chang D, Dirks B, Tsaban G, Kaplan A, Rinott E, et al. Wolffia globosa–mankai plant-based protein contains bioactive vitamin b12 and is well absorbed in humans. Nutrients 2020;12:1–17.

13. Zelicha H, Kaplan A, Meir AY, Tsaban G, Rinott E, Shelef I, Tirosh A, Brikner D, Pupkin E, Qi L, et al. The Effect of Wolf fi a globosa Mankai , a Green Aquatic Plant , on Postprandial Glycemic Response : A Randomized Crossover Controlled Trial. Diabetes Care 2019;42:1–8.

14. Tsaban G, Meir AY, Rinott E, Zelicha H, Kaplan A, Shalev A, Katz A, Rudich A, Tirosh A, Shelef I, et al. The effect of green Mediterranean diet on cardiometabolic risk ; a randomised controlled trial. 2020;1–8.

15. Matthews DR, Hosker JP, Rudenski AS, Naylor BA, Treacher DF, Turner RC. Homeostasis model assessment: insulin resistance and β-cell function from fasting plasma glucose and insulin concentrations in man. Diabetologia 1985;28:412–9.

16. Bu G. Apolipoprotein E and its receptors in Alzheimer’s disease: pathways, pathogenesis and therapy. Nat Rev Neurosci 2009;

17. Yi L, Wu T, Luo W, Zhou W, Wu J. A non-invasive, rapid method to genotype late-onset Alzheimer’s disease-related apolipoprotein E gene polymorphisms. Neural Regeneration Research 2014;9:69–75.

18. Heister D, Brewer JB, Magda S, Blennow K, McEvoy LK. Predicting MCI outcome with clinically available MRI and CSF biomarkers. Neurology 2011;77:1619–28.

19. Ross DE, Ochs AL, Desmit ME, Seabaugh JM, Havranek MED. Man versus machine part 2: Comparison of radiologists’ interpretations and neuroquant measures of brain asymmetry and progressive atrophy in patients with traumatic brain injury. Journal of Neuropsychiatry and Clinical Neurosciences 2015;27:147–52.

20. Silbert LC, Quinn JF, Moore MM, Corbridge E, Ball MJ, Murdoch G, Sexton G, Kaye JA. Changes in premorbid brain volume predict Alzheimer’s disease pathology. Neurology 2003;61:487–92.

21. Madhyastha T, Mérillat S, Hirsiger S, Bezzola L, Liem F, Grabowski T, Jäncke L. Longitudinal reliability of tract-based spatial statistics in diffusion tensor imaging. Human Brain Mapping John Wiley and Sons Inc.; 2014;35:4544–55.

22. Wassenaar TM, Yaffe K, van der Werf YD, Sexton CE. Associations between modifiable risk factors and white matter of the aging brain: insights from diffusion tensor imaging studies. Neurobiology of Aging Elsevier Inc.; 2019;80:56–70.

23. Bakun Emesh T, Garbi D, Kaplan A, Zelicha H, Yaskolka Meir A, Tsaban G, Rinott E, Meiran N. Retest Reliability of Integrated Speed–Accuracy Measures. Assessment SAGE Publications Inc.; 2021;

24. Wishart DS, Feunang YD, Marcu A, Guo AC, Liang K, Vázquez-Fresno R, Sajed T, Johnson D, Li C, Karu N, et al. HMDB 4.0: The human metabolome database for 2018. Nucleic Acids Research 2018;46:D608–17.

25. Rothwell JA, Perez-Jimenez J, Neveu V, Medina-Remón A, M’Hiri N, García-Lobato P, Manach C, Knox C, Eisner R, Wishart DS, et al. Phenol-Explorer 3.0: A major update of the Phenol-Explorer database to incorporate data on the effects of food processing on polyphenol content. Database 2013;2013:1–8.

26. Ihaka R, Gentleman R. R: A Language for Data Analysis and Graphics. Journal of Computational and Graphical Statistics 1996;5:299–314.

27. Erickson KI, Voss MW, Shaurya R, Basak C, Szabo A, Prakash RS, Basak C, Szabo A, Chaddock L, Kim JS, et al. Exercise training increases size of hippocampus and improves memory. Proceedings of the National Academy of Sciences [Internet] 2011;108:3017–22. Available from: http://www.pnas.org/cgi/doi/10.1073/pnas.1015950108
